# *Pterocarpus soyauxii* (Fabaceae) aqueous extract to prevent neuropsychiatric disorders associated with menopause by triggering ROS-dependent oxidative damage and inhibiting acetylcholinesterase, GABA-transaminase, and monoamine oxidase A: *In vitro*, *in vivo*, and *in silico* approaches

**DOI:** 10.1016/j.heliyon.2024.e33843

**Published:** 2024-06-28

**Authors:** Pascal Emmanuel Owona, Yolande Sandrine Mengue Ngadena, Danielle Claude Bilanda, Madeleine Chantal Ngoungouré, Lohik Mbolang Nguegan, Ronald Bidingha A Goufani, Rivaldo Bernes Kahou Tadah, Michel Noubom, Armand Fils Ella, Yannick Carlos Tcheutchoua, Bruno Dupon Ambamba Akamba, Paule Cynthia Bouguem Yandja, Paulin Keumedjio Teko, Paul Desire Dzeufiet Djomeni, Pierre Kamtchouing

**Affiliations:** a Department of Animal Biology and Physiology, Laboratory of Animal Physiology, Faculty of Science, University of Yaoundé 1, P.O. Box 812 Yaoundé, Cameroon; b Neurosciences and psychogerontology axis, Laboratory of Development and Maldevelopment, Department of Psychology, Faculty of Arts, Letters, and Social Science, University of Yaoundé 1, P.O. Box. 755 Yaoundé, Cameroon; c Department of Biological Sciences, Faculty of Medicine, University of Dschang, P.O. Box. 67, Dschang, Cameroon; d Department of Biochemistry, Laboratory of Pharmacology and Toxicology, Faculty of Science, University of Yaoundé 1, P.O. Box 812 Yaoundé, Cameroon

**Keywords:** Menopause, Neurological disorders, *Pterocarpus soyauxii*, Neuroprotective, Antioxidant, Anti-inflammatory

## Abstract

*Pterocarpus soyauxii* (PS) is traditionally used in Cameroon medicine to alleviate postmenopausal symptoms. Previous research has shown that it has tissue-selective potential and estrogen-mimetic effects on vaginal atrophy. Phytoestrogens like 7-*O*-acetyl formononetin, khrinone A, and 3′,5′-dimethoxy-4-stilbenol were found in its water extract by UHPLC, but there is no evidence of its effects on neurological disorders linked to post-menopause (ND-PO). The study aimed to investigate the phytochemical profile of PS aqueous extract, assess its neuroprotective potential in rats, and explore possible underlying pathways. We used colorimetric assays to study the phytochemical profile of PS extract. Effects of the extract on behavioral parameters, neuronal signaling, and integrity in an 84-day ovariectomized rat model. Molecular docking was performed to assess the ability of 7-*O*-acetyl formononetin, an isoflavone contained in PS, to cross the BBB and its binding affinity to the active sites of AChE, MAO-A, and GABA-T. Besides, the anti-AChE/BChE, antioxidant, and anti-inflammatory effects of PS were assessed by *in vitro* tests. PS aqueous extract contains polyphenols (656.58 ± 9.18 mgEAG/100gMS), flavonoids (201.25 ± 5.52 mgEQ/100gDW), and tannins (18.42 ± 1.25 mg/100gDW). It slows down anxiety, depressive disorders, cellular disorganization, and neuronal death in the hippocampus, dentate gyrus, and neocortex. *In silico* modeling was a powerful tool to assess the 7-*O*-acetylformononetin's ability to cross the BBB and strongly bind and inhibit AChE, MAO-A, and GABA-T. Thus, by combining GABAergic, cholinergic, and serotoninergic modulation, PS aqueous extract also possesses remarkable anti-AChE/BChE *in vitro* and induces antioxidant and anti-inflammatory potential in macrophages. Such estromimetics, antioxidant, anti-inflammatory, cholinergic, and monoaminergic modulators represent promising activities to develop neuroprotective drugs with optimal therapeutic profiles for menopausal women.

## 1 Introduction

Menopause is a physiological state marked by the end of the reproductive period in women, commonly around 50–55 [1]. The drastic fall in estrogen levels associated with the loss of ovarian follicular activity during menopause is associated with a range of neuropsychiatric symptoms, such as long- and short-term memory loss, anxiety, and depression [2]. According to the World Health Organization [3], the number of postmenopausal women worldwide is rising, and women are living longer. Indeed, a woman turning 60 in 2019 might anticipate living another 21 years on average worldwide. Many hallmark symptoms characterize menopause. Among them, low levels of estrogen promote the onset of neurological disorders, especially forgetfulness, sleep disturbances, and altered mood. Epidemiological data show that almost 38 % and 36 % of menopausal women have experienced anxiety and depressive disorders at least once [4]. In addition, postmenopausal women are highly vulnerable to memory problems, which can reach 70 % by the age of 65 [5]. Estrogen deficiency is associated with neuronal loss, apoptosis of hippocampal neurons, cognitive dysfunction, and a decrease in cerebral concentrations of monoamine (MAO) like serotonin (5-HT), acetylcholine (ACh), and γ-amino-butyric acid (GABA), alterations that would explain the onset of cognitive problems in menopause [6,7]. Among others, oxidative stress and inflammation are critical factors in the pathogenesis of neuropsychiatric disorders during menopause [[8], [9], [10]].

Cerebral oxidative stress during menopause is often induced by the activation of NADPH oxidase, leading to membrane peroxidation [11]. Postmenopausal neuroinflammation is also linked to the activation of microglial cells, which are characterized by nitric oxide and 5-lipoxygenase production and low alkaline phosphatase activity [9]. These two factors are thought to induce neuronal damage, resulting in cognitive impairment and the development of neurodegenerative diseases with age [12]. The management of these disorders is based on Hormone Replacement Therapy (HRT) [13]. Nevertheless, HRT promotes the onset of side effects such as the development of breast cancer [1,14]. Thus explaining the eagerness of scientists to seek new molecules to treat nervous disorders in postmenopausal women. Pleiotropic actions of estrogen-like compounds such as phytoestrogens are reported to regulate neurological disorders due to menopause [15,16]. Studies have shown that the leaves of *Pterocarpus soyauxii* (PS) inhibit acetylcholinesterase (AChE) activity [17], and according to Saslis-Lagoudakis et al. [18], *Pterocarpus* species-genus is used to treat nervous disorders.

Previous studies reported the estrogenic activities of PS heartwood aqueous extract [19], and ethnobotanical investigations show that PS is used to manage menopausal symptoms in the Centre Region of Cameroon [20]. The phytochemical study revealed through UHPLC that PS contains secondary metabolites like 7-*O*-acetyl formononetin, Khrinone A, 3′,5′-dimethoxy-4-stilbenol, and linoleic acid [19]. As for the various pharmacological potentials of PS on vaginal dryness and anti-AChE, does it also have neuroprotective potential? At present, knowledge of the neuroprotective potential of PS is still scarce, as are the pathways involved. It is hoped that our study will allow further insights into the exploration of a multitarget drug derived from PS against menopause-related disorders. The main objective of the present study was to 1) investigate the phytochemical profile of PS aqueous extract, 2) assess the neuroprotective potential of PS aqueous extract in rats, and 3) explore possible underlying pathways. It is hoped that our study will allow further insights into the exploration of a multitarget drug derived from PS against menopause-related disorders.

## 2 Material and methods

### 2.1 Materials, chemicals, and reagents

Diazepam (Valium® 10 mg) and 17-β Estradiol valerate (Progynova® 2 mg) were purchased from DELPHRAM (Lille, France). Donepezil Aricept was purchased from FAREVA (Belgium). The following chemicals were obtained from Sigma Ald Louis in MO, USA: Aluminum chloride, DTNB (5,5-dithiobis(2-nitrobenzoic acid)), adrenaline (C₉H₁₃NO₃), sulfanilamide (C_6_H_8_N_2_O_2_S), N-1-naphthylene diamine dichloride (C_12_H_16_Cl_2_N_2_), phosphoric acid (H₃PO₄), hydrogen peroxide (H_2_O_2_) hydrolyzing, potassium dichromate (K_2_Cr_2_O_7_), trichloroacetic acid (C_2_HCl_3_O_2_), and thiobarbituric acid (C_4_H_4_N_2_O_2_S), chlorhydric acid (HCl), nitro blue tetrazolium, potassium hydroxide (KOH), sodium chloride (NaCl), iron sulfate (FeSO4), Triton X-100, *p*-nitrophenyl phosphate, dimethyl sulfoxide (DMSO), sodium diclofenac, sulfuric acid, sodium phosphate, ammonium molybdate.

### 2.2 Plant material, extraction, and phytochemical profiling

Samples of PS heartwoods were collected from Ngomedzap, Centre Region, Cameroon, during the rainy season. The collected heartwoods were air- and dark-dried before finely grounding. The aqueous extract of PS was performed according to Mengue et al. [19]. A phytochemical analysis of PS aqueous extract was carried out using quantitative assays. The flavonoid content of PS extract was determined by the reaction of quercetin with aluminum chloride and sodium acetate, according to Zhishen et al. [21]. Total polyphenols were assessed spectrophotometrically using the Folin-Ciocalteu reagent. Tannins were determined using the method described by Broadhurst and Jones [22]. using acidified vanillin and tannic acid as the standard.

### 2.3 Animal species and ethical statement

The Animal Welfare Act was complied with in the general care of the experimental animals used during the present study. All animal tests were conducted following the Animals (Scientific Procedures) Act of 1986 and the EU Directive 2010/63/EU for experiments on animals. Randomization procedures were performed during the research study under the ARRIVE 2.0 criteria (https://arriveguidelines.org/arrive-guidelines). Healthy female albinos Wistar rats (8–10 weeks old) weighing 120–130 g and male BALB-C mice (8–10 weeks old) were supplied by the Animal Physiology Laboratory, University of Yaoundé 1 (Cameroon) production facility. All rats were housed in clean plastic cages at room temperature (natural cycle). They had *ad-libitum* access to tap water and soy-free rat chow for a week before experiments. All experiments in this study were conducted under the principles and procedures of the European Union on Animal Care (CEE Council 86/609) guidelines adopted by the Cameroon Institutional National Ethics Committee, Ministry of Scientific Research and Technology Innovation (Reg. number FWA-IRD 0001954).

### 2.4 Animal model establishment and experimental groups

Neurological disorders associated with post-menopause (ND-PO) were induced after 84 days of bilateral ablation of the ovaries in female rats. After anesthesia by intraperitoneal injection of diazepam (10 mg/kg) and ketamine (50 mg/kg), ablations of the ovaries were carried out according to the Li et al. [23] protocol. Briefly, a portion of the lower back was shaved, and then the area was disinfected with a piece of cotton soaked in 95 % alcohol. A thin transverse incision was made in the skin over the shaved area. The peritoneum was incised using fine forceps, and the opening was maintained to allow extraction of the ovary mass. The ovary was separated from the uterine horn using fine scissors. The fatty mass was then reintroduced, and the peritoneum was sutured. Finally, the skin was sutured, and the wound was dressed with Betadine and penicillin ointment for two weeks.

**Fig. 1:**
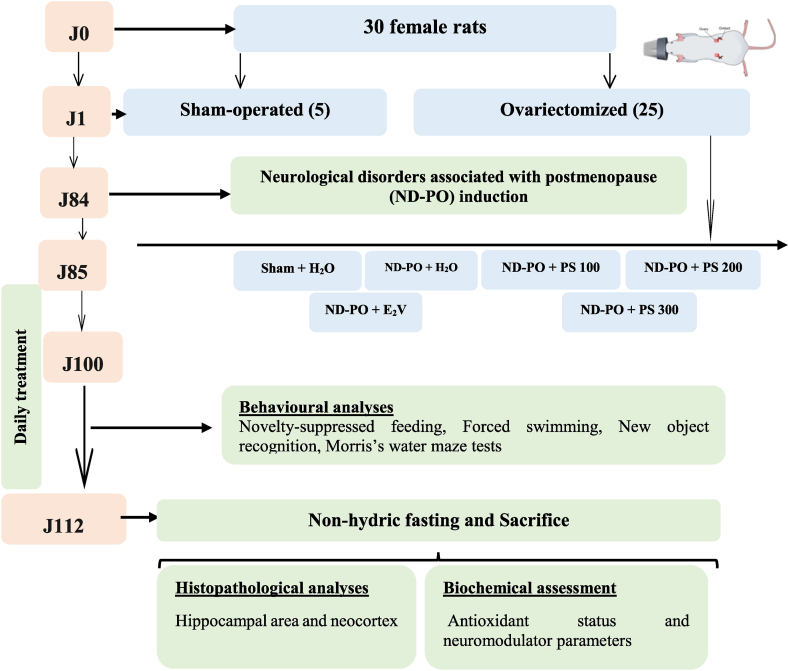
Chart flow of *in vivo* experiments. **SHAM + H**_**2**_**O**: sham-operated rats receiving distilled water (10 mL/kg); **ND-PO + H**_**2**_**O**: ovariectomized rats with postmenopausal disorders and receiving distilled water (10 mL/kg); **ND-PO + E**_**2**_**V**: ovariectomized rats with postmenopausal disorders and receiving valerate estradiol (1 mg/kg); **ND-PO + PS 100, ND-PO + PS 200, ND-PO + PS 300**: ovariectomized rats with postmenopausal disorders and receiving *P. soyauxii* aqueous extract at 100, 200, and 300 mg/kg respectively.

Thirty rats were randomly divided into sham (05) and ovariectomized (Ovx) (25) groups and administered daily *per os* for 28 days. Ovx rats received the vehicle (10 mL/kg), estradiol valerate (E_2_V) at 1 mg/kg, and extract at 100, 200, and 300 mg/kg, respectively. A sham-operated group receiving the vehicle (10 mL/kg) is a control. Between 100 and 112 days after surgery, the animal's behavior was assessed via the novelty-suppressed feeding test, the forced swim test, the new object, and Morris's water maze recognition tests. The study flow chart is shown in Fig. 1. Ultimately, they were sacrificed, and the arteriovenous blood was collected and centrifuged at 3500 rpm for 15 min. The serum was collected, sampled, and used to evaluate protein, calcium, and magnesium concentrations. A 0.4 g of the hippocampal region was homogenized in 2 mL of Tris buffer. The homogenate obtained was centrifuged at 3500 rpm for 30 min. The supernatant was collected and sampled for the evaluation of some markers of the oxidative status (MDA, GSH, and nitrites), the assessment of gamma-aminobutyric acid (GABA), acetylcholine (ACh), serotonin (5-HT), proteins, calcium, and magnesium levels, as well as the activity of GABA-transaminase (GABA-T). The remaining part of the hippocampal region was used for histological analysis.

### 2.5 Behavioral tests

Behavioral studies lasted 12 days and were evaluated during treatment administration for ND-PO. The ANY-maze video tracking system (Stoeltimg, Wood Dale, IL, USA) recorded and analyzed all testing with a Logitech camera.

#### 2.5.1 Novelty-suppressed feeding test

Aimed to measure anxiety in rats, this test evaluates how long it takes for the animal to approach and consume an amount of known food in a new and unpleasant setting. Alena et al. [24] established that after a non-hydric fast of 24 h, 30 min before the test, each animal was removed from its regular cage and put in a holding cage. A 60-W incandescent lamp was positioned 1.2 m above the rats in a 50 by 50 by 20-cm open arena. A tiny food pellet weighing 10 g was placed in the center of an open arena with a white filter paper measuring 12.5 cm in diameter.

#### 2.5.2 Forced swimming test

To determine rodent depression, a rat was placed in a water tank (50 cm high, 30 cm wide) and kept at room temperature according to the Erika et al. [25] procedure. An animal was immobile if it floated horizontally and made only tiny amplitude movements to keep its head above water. The pre-test and test phases, separated by a day, comprised the two test stages. Throughout the test, the time spent swimming, ascending, and inactive were timed and recorded in seconds.

#### 2.5.3 New object recognition test

The purpose of this test was to assess rodents' episodic memory. It was performed following the procedure laid out by Andrzej et al. [26]. Concretely, the animal was taught for 5 min in an open arena (50 x 50 × 20 cm). For 5 min, participants in the program are free to explore two identical items. The animal is removed from the arena and put back in its customary cage for an hour. The same animal was returned to the open arena for the test's second phase. The animal could spend over 5 min investigating two things for that second phase. One of the two identical and well-known things the animal has previously studied was changed to a new object.

#### 2.5.4 Morris's water maze test

The Morris water maze test is one of rodents' most commonly used behavioral tests to measure spatial learning. According to Morris's [27] original description of the paradigm, this task was modified for use with rats. The water maze was a rectangular pool with a circumference of 150 cm and a depth of 60 cm divided into four quadrants. It contained 40 cm of opaque, room-temperature water. One quadrant had an escape platform in the center, 1.0 cm below the water's surface, and evenly spaced between the pool's sidewall and center. The only way out of the sea was on a platform in the same quarter. Around the pool's edge, three distinct starting sites for rats were set up. The beginning point differed on the four training days since each start place was utilized once in a supposedly random order.

During each training session, the test animal has 5 min to swim in the water to locate the hidden platform. The animal is rescued and placed on the platform if it cannot find it within 5 min. After 5 min on the platform, each animal returns to its cage. Every day, every animal receives five training sessions. The animal has to learn to locate the platform by using the visual signals surrounding the pool, as it is hidden. The escape latency, or the time it takes to discover the concealed platform, usually decreases with training. Rats were given a probe test, in which the platform was taken out of the pool, and the rodent was free to look for it, usually for 60 s, to distinguish between spatial and non-spatial strategies.

### 2.6 Biochemical assays

Brain calcium and magnesium levels were assessed using commercial diagnostic kits from Biolabo and Randox. Calcium concentration was determined by its reaction with o-cresol phthalein complexion by the protocol of Clarck et al. [28]. The magnesium level was determined by its reaction with xylidyl blue complexion by the protocol of Treiz [29]. The activity of GABA-transaminase (GABA-T) and the gamma aminobutyric acid (GABA) concentration in brain homogenate were determined using protocols described by Nayak et al. [30]. Serotonin (5-HT) and acetylcholine (ACh) levels were determined using methods described, respectively, by Yoshitake et al. [31] and Hestrin [32]. Malondialdehyde (MDA) and reduced glutathione (GSH) in brain homogenate were determined using the procedures described by Wilbur [33] and Ellman [34], respectively, while the nitrite content was determined using the method described by Green et al. [35].

### 2.7 Histological analysis and neuron counting

The hippocampus area was fixed in 10 % buffered formalin for two weeks, and then coronal sections were cut out and dried using a croissant-shaped alcohol gradient (70 %, 80 %, 90 %, and 100 % in three baths). The tissues were clarified using xylene in two baths, each for 1 h and 30 min, and then waxed with liquid paraffin at 60 °C for 5 h before sectioning. The sliced tissues were baked, dewaxed, and stained with Mayer's hematoxylin for 5 min, water-washed, and stained with 1 % water-soluble eosin for another 5 min. Then, the tissues were washed and sealed, and pictures were taken of them under a microscope. The number of hippocampus neurons in the CA1 and CA3 areas was counted using microphotography taken with a light microscope (Leitz Wetzlar Germany 513) attached to a digital camera (Celestron 44421) connected to a computer. The pictures were transmitted and analyzed using the ImageJ program. The neocortex's external granular layer (lamina granularis externa), the second layer of the neocortex from the dorsal periphery, was captured.

### 2.8 *In vitro* antioxidant potential of PS

In experiments, the percentage of inhibition was determined by the following formula:

$$\%\;\text{Inhibition}=\frac{\left(\text{OD}\;\text{control}-\text{OD}\;\text{sample}\right)}{\text{OD}\;\text{control}}\;x\;100$$

#### 2.8.1 Total antioxidant capacity investigation

The antioxidant capacity of *P. soyauxii* heartwood extract was measured using the method described by Prieto et al. [36]. To summarize, 0.3 mL of plant extract was mixed with 3 mL of a reagent solution containing 0.6 M sulfuric acid, 28 mM sodium phosphate, and 4 mM ammonium molybdate. The tubes were tightly sealed and incubated at 95 °C for 90 min. Once cooled, the absorbance of the solutions was measured at 695 nm against a blank sample, which contained 3 mL of the reagent solution and 0.3 mL of methanol and was incubated under the same conditions as the sample. The total antioxidant capacity was expressed in milligrams of ascorbic acid equivalents per gram of dry matter (mg EAA/g DW).

#### 2.8.2 Anti-lipoperoxidative activity of PS

The anti-lipoperoxidative activity of *P. soyauxii* heartwood extract was tested using the Moukette et al. [37] method. To carry out the test, 300 μL of extract (concentrations of 5–6.25-7.5-8.75 and 10 mg/mL) was added to 500 μL of liver homogenate. Then, 100 μL NaCl and lipid peroxidation were initiated by adding 100 μL iron sulfate. The mixture was incubated at 37 °C for 30 min. After that, 1000 μL of thiobarbituric acid (1 %)/HCl (10 %) at equal volume was added to the solution, followed by 1000 μL of ascorbic acid. The final mixture was heated to 80 °C for 20 min in a water bath, cooled, and then centrifuged at 1500 g for 10 min. Optic densities were read at 532 nm. The control was carried out without adding extract. The results were expressed as percentages of lipoperoxidation inhibition.

#### 2.8.3 Effects of PS on NADPH oxidase activity

To determine the effects of PS on NADPH oxidase (NADPH-o) activity, a protocol previously described by Manosroi et al. [38] was followed. 150 μL of cells (at a concentration of 104 cells/mL) were mixed with 50 μL of PS extract and incubated at 37 °C and 5 % CO_2_ for 3 h. Following this, 50 μL of Saccharomyces cerevisiae and 50 μL of nitro blue tetrazolium (NBT) were added to the medium and incubated at 37 °C and 5 % CO_2_ for 1 h. The supernatant was then removed, and the pellets were dissolved in 20 μL of methanol and left at room temperature for 10 min. Next, 50 μL of KOH 2 M and 50 μL of dimethyl sulfoxide (DMSO) were added to the medium. The absorbance was measured at 570 nm, and the results were expressed as the percentage of NADPH-o activity inhibition.

### 2.9 *In vitro* anti-inflammatory activities

#### 2.9.1 Macrophage isolation and incubation

To isolate primary mouse macrophages, we started by injecting 0.5 mL of a 2 % starch solution in the intraperitoneal area of mice to elicit inflammation [39]. Four days later, mice were sacrificed by cervical dislocation. We then injected 20 mL of PBS buffer (0.1 M, pH 7.4) into the peritoneal cavity of rats and 5 mL in mice to collect macrophages. After massaging the abdominal cavity, we slowly aspirated the injected buffer. The macrophage-containing solution was placed in 15-mL Falcon tubes and kept on ice. The fluid was centrifuged (3000 rpm, 4 °C, 10 min), and the supernatant was discarded. The cell-containing pellet was recovered, and red blood cells were removed through osmotic shock [40]. We suspended the cells in 1 mL of a hypotonic 0.05 M NaCl solution for 1 min and then restored isotonicity by adding 1 mL of 0.25 M NaCl. The mixture was centrifuged again (3000 rpm, 4 °C, 10 min), and the resulting pellet containing mostly macrophages was suspended in 2 mL of Dulbecco's Modified Eagle medium (DMEM) and kept on ice. The cells were then distributed in different wells at 104 cells/mL. The test and positive control wells contained 150 μL of cells mixed with 50 μL of Saccharomyces cerevisiae (250 μg/mL). The blank wells had 150 μL of cells combined with 50 μL of DMEM. The microplate was then incubated for 1 h at 37 °C (5 % CO_2_). After that, we added 50 μL of extract at different concentrations (0.1, 1, 10, 50, and 100 μg/mL) to the test wells, 50 μL of DMEM to the positive control wells, and 50 μL of baicalin to the standard. The microplate was incubated for 3 h at 37 °C (5 % CO_2_). We used the supernatants for nitric oxide assays and the pellets for assays of alkaline phosphatase and lipoxygenase activities, Bovine Serum Albumin (BSA) denaturation, and nitric oxide (NO) production.

#### 2.9.2 Effect of PS on alkaline phosphatase activity

The experiment was conducted using Sun et al. [41] method. To start, the pellet cells were solubilized using 25 μL Triton X-100. Then, 50 μL of a solution containing 10 mM *p*-nitrophenyl phosphate and 50 μL of glycine buffer (0.1 M, pH 9.0) were added. The mixture was incubated for 30 min at 37 °C. 100 μL of NaOH buffer (0.2 M, pH 12) was added to stop the reaction. The absorbance was then measured at 405 nm, and the percentage change in lysosomal enzyme activity was calculated using the Suzuki et al. [42] method.

#### 2.9.3 Effect of PS on NO production

This test is based on the Griess diazotization reaction first described in 1879. Sulphanilamide and naphthyl ethylene diamine dihydrochloride (NED) compete for nitrate in an acidic reaction. During phagocytosis, nitric oxide forms complexes with Griess' reagent, producing a yellow-colored compound that absorbs at 550 nm. In this experiment, we used the supernatants obtained earlier. To start, 100 μL of supernatant was mixed with 100 μL of Griess reagent (1 % Sulphanilamide, 0.1 % naphthyl ethylene diamine dihydrochloride in 2.5 % v/v phosphoric acid). The mixture was incubated at room temperature for 10 min, and the absorbance was measured at 550 nm. The amount of nitrite was then measured using the standard sodium nitrate curve. Finally, the percentage inhibition of nitric oxide production was calculated.

#### 2.9.4 Effect of PS on BSA denaturation

The study aimed to investigate the impact of *P. soyauxii* on BSA denaturation using Elias and Rao's method [43]. In each test tube, 450 μL of BSA and 50 μL of extract were added. For standard tubes, 50 μL of sodium diclofenac was used instead of extract. In the control tubes, 50 μL of extract or diclofenac was mixed with 450 μL of distilled water. The tubes were incubated for 20 min at 37 °C and 30 min at 57 °C. Then, 2500 μL of 0.1 M phosphate-buffered saline, pH 7.4, was added to each tube, and the absorbance was measured at 660 nm. The percentage inhibition of protein denaturation was calculated.

#### 2.9.5 Effect of PS on 5-lipoxygenase activity

The impact of *P. soyauxii* on 5-lipoxygenase activity was measured using the method previously outlined by Yougbaré-Ziebrou et al. [44]. In each test tube, 300 μL of Saccharomyces cerevisiae suspension (250 μg/mL) was added, except in the negative control where culture medium was used instead. The tubes were then incubated at 37 °C with 5 % CO_2_ for 1 h. After that, 50 μL of extract was added to the test tubes, and 50 μL of ascorbic acid, acetylsalicylic acid, and baicalin were added to the standard tubes. In comparison, 50 μL of medium was added to the control tubes. The tubes were then incubated for an additional 3 h. After the incubation, each tube was centrifuged at 2000 rpm for 10 min at 4 °C, and the supernatant was discarded. The pellet containing the cells was then resuspended in 50 μL of Triton X-100, and the tubes were shaken for 2 min. Lastly, 1000 μL of linoleic acid (125 μM) was added to each tube, and the tubes were incubated for 30 min. The optical density of the supernatant was then measured at 234 nm, and the percentage inhibition of the enzyme's activity was calculated.

### 2.10 *In vitro* assays of anti-AChE/BChE activities of PS

The inhibition of AChE (acetylcholinesterase) and BChE (butyrylcholinesterase) activities were tested in a spectrophotometric cuvette three times using the following steps: 400 μL of DTNB (1 mmol/L), 50 μL of AChE or BChE (20 % rat brain homogenate), 450 μL of PBS, and 75 μL of extract solution (0–5 mg/mL) prepared in DMSO. The reaction was initiated by 125 μL of acetylthiocholine iodide and butyrylthiocholine iodide (75 mmol/L). Donepezil (0–1000 μg/mL) was used as a reference. After 5 min of incubation at 37 °C, the absorbance was measured at 412 nm against the blank in the spectrophotometer [45]. The inhibition percentages were then calculated.

### 2.11 *In silico* molecular docking

#### 2.11.1 Software used

The following software packages were downloaded from various URLs: Python 2.7 from www.python.com, Cygwin (a data store) c:\program, and Python 2.5 simultaneously from www.cygwin.com. Molecular Graphics Lab Tools (MGL) and AutoDockTools-1.5.7 from www.scripps.edu; Discovery Studio Visualizer 2.5.5 from www.accelerys.com; and ChemDraw from www.clubic.com. Additionally, online smile scoring was performed using ht tps://cactus.nci.nih.gov/translate/[46,47].

#### 2.11.2 Preparation of the target enzymes

The crystal structure of three proteins, namely the mouse acetylcholinesterase (5HCU), the MOA A (4ZSW), and the GABA aminotransferase (2Z5X) were downloaded from the Research Collaboratory for Structural Bioinformatics (RCSB) protein database. Next, the proteins were prepared using AutoDock tools, which involved adding all hydrogen atoms to the macromolecule, a necessary step to calculate partial atomic charges correctly. Three-dimensional affinity grids were generated for each protein's active site, namely the Catalytic triad (Ser 203, His 447, Glu 334), Oxyanion hole (Gly121, Gly 122, Ala 204), Acyl pocket (Phe295, Phe297, Trp 236, Phe338), Anionic site (Trp86), and Peripheral site (Tyr72, Asp 74, Tyr124, Trp 286, Tyr341) [48] of size 60 × 58 × 68 Å with a spacing of 0.375 Å for the AChE protein, the GABA aminotransferase active site (Arg 192; Lys 203; Glu 265; Arg 445) of size 40 × 48 × 48 Å with a spacing of 0.375 Å, and the MOA A active site (Lys 305; Trp 397; Tyr 407; Tyr 444 [48]) of size 58 × 46 × 72 Å with a spacing of 0.375 Å [49]. These affinity grids were centered on the geometric center of the respective proteins. They were calculated for each of the following atom types: HD, C, A, N, OA, and SA, representing all possible atom types in a protein [50].

### 2.12 Preparation of the identified ligand of PS

The 2D structures of the PS, donepezil, moclobemide, and vigabatrin compounds were drawn using ChemDraw software. The 3D structures were obtained using ChemDraw 3D software, and energy minimization was performed using the MM2 force field and saved in PDB format with the same software.

#### 2.12.1 *In silico* inhibition of AChE, MAO-A, and GABA-T

The Lamarckian genetic algorithm (LGA), a hybrid of a genetic algorithm and a local search algorithm, was used for ligand conformation search. This algorithm first constructs a population of individuals (genes), each having a different random conformation of the anchored molecule. Each individual is then mutated to acquire a slightly different translation and rotation, and the local search algorithm then performs energy minimizations on a user-specified proportion of the population of individuals. The individuals with the lowest resulting energy are transferred to the next generation, and the process is repeated. The algorithm is called Lamarckian because each new generation of individuals is allowed to inherit the local search adaptations of its parents. Gasteiger charges are calculated for each atom of the macromolecule in AutoDock 4.2 instead of the Kollman charges used in previous versions of this program [51]. The docking calculation in AutoDock 4.2 was performed using the refined protein and the desired ligand in pdb format. The.glg file executes the AutoGrid calculation. The.dlg file runs AutoDock. Finally, ten different conformations were studied for a single compound. For each ligand, the ten best docking simulations were obtained against the target molecule [52]. The results were visualized using the Discovery Studio Visualizer.

### 2.13 Data analysis

The data analysis and IC50 values were conducted using GraphPad Prism 8.0.1 software. The data are presented as mean ± Standard Error on Mean (SEM). When needed, statistical comparisons between different experimental groups were calculated by one-way ANOVA and the Tukey post hoc test. A p-value below 0.05 was considered statistically significant. Principal component analysis was performed using R 4.3.1 software.

## 3 Results

### 3.1 Quantitative phytochemical assays of PS

The concentrations of secondary metabolites that can specify the plant profile were determined to assess the phytochemical composition of PS aqueous extract. The water extract of PS was analyzed by colorimetric assays, revealing the bioactive compounds' presence (Table 1). The results expressed per 100g of dry weight (DW) showed that PS has appreciable concentrations of polyphenols (656.58 ± 9.18 mgEAG), flavonoids (201.25 ± 5.52 mgEQ), and tannins (18.42 ± 1.25 mg).

**Table 1:** Quantitative phytochemical screening of aqueous extract of PS.

Secondary metabolites	Aqueous extract of *P. soyauxii*
**Polyphenols** (mgEAG/100 gDW)	656.58 ± 9.18
**Flavonoids** (mgEQ/100 gDW)	201.25 ± 5.52
**Tannins** (mg/100 gDW)	18.42 ± 1.25

Each value represents concentration and is expressed as mean ± SEM; n = 3.

**mgEAG;** milligram equivalent of gallic acid**, mgEQ;** milligram equivalent of quercetin, **gDW**: gram of dry weight.

### 3.2 Protective outcomes of PS on Ovx-induced behavioral disorders *in vivo*

#### 3.2.1 Anxiolytic effects of PS

**Fig. 2:**
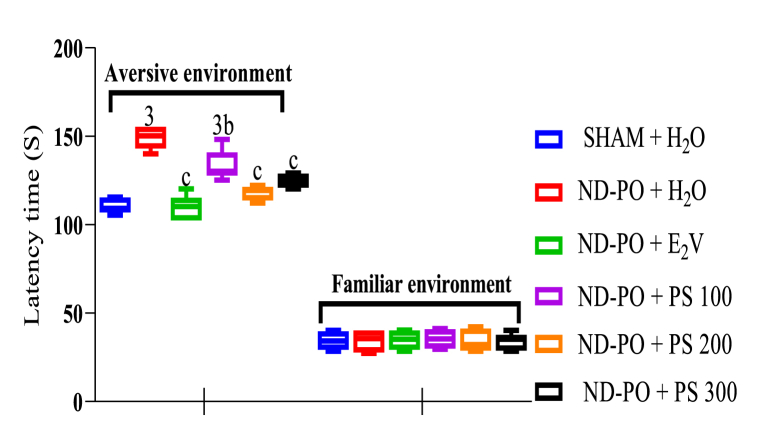
Determination of anxiolytic potential of PS extract during NSF. Each bar represents mean ± MSE; n = 5; ^3^p < 0.001, a significant difference compared to sham-operated control; ^b^p < 0.01; ^c^p < 0.001, a significant difference compared to ND-PO control. **SHAM + H**_**2**_**O**: sham-operated rats receiving distilled water (10 mL/kg); **ND-PO + H**_**2**_**O**: ovariectomized rats with postmenopausal disorders and receiving distilled water (10 mL/kg); **ND-PO + E**_**2**_**V**: ovariectomized rats with postmenopausal disorders and receiving valerate estradiol (1 mg/kg); **ND-PO + PS 100, ND-PO + PS 200, ND-PO + PS 300**: ovariectomized rats with postmenopausal disorders and receiving *P. soyauxii* aqueous extract at 100, 200, and 300 mg/kg respectively.

Ovariectomy led to a significant increase in anxiety, as evidenced by elevated latency time to bite the food (LTB) in the aversive environment (p < 0.001) compared to the sham-operated group during the novelty-suppressed feeding test (NSF). Administration of PS extract at a dose of 100 mg/kg significantly (p < 0.01) reduced LTB compared to the ND-PO control group. Likewise, the extract at doses of 200 and 300 mg/kg in the same way as estradiol valerate resulted in a significant decrease (p < 0.001) in latency time as compared to the ND-PO control group (Fig. 2).

#### 3.2.2 Ameliorative impact of PS on depressive behavior

The ablation of the ovaries induced severe depressive behavior, characterized by a significant decrease (p < 0.001) in both swimming and climbing time during the forced swimming test (FS). Likewise, It increased the immobility time (p < 0.01) compared to the sham control. *P. soyauxii* aqueous extract at 200 mg/kg significantly reduced immobility time and significantly increased swimming and climbing compared to ND-PO control (Table 2). The reduction of immobility and climbing times at 200 mg/kg was respectively from 164.86 ± 3.32 s and 42.72 ± 1.78 s to 129.35 ± 3.89 s and 65.36 ± 4.49 s compared to the ND-PO control. At the dose of 300 mg/kg, the extract caused a significant decrease (p < 0.001) in immobility time and a significant increase in swimming (p < 0.05) and climbing time (p < 0.01).

**Table 2:** Determination of antidepressant potential of PS extract during FS.

Parameters Groups	Immobility (s)	Swimming (s)	Climbing (s)
SHAM + H_2_O	132.28 ± 3.57	106.27 ± 5.12	59.71 ± 3.18
ND-PO + H_2_O	164.86 ± 3.32^b^	81.29 ± 1.97^b^	42.72 ± 1.78^a^
ND-PO + E_2_V	131.73 ± 5.70^c^	99.43 ± 5.32^a^	56.46 ± 2.70^b^
ND-PO + PS 100	148.79 ± 2.52^b^	84.03 ± 3.43^a^	45.57 ± 2.56
ND-PO + PS 200	129.35 ± 3.89^c^	103.65 ± 1.90^b^	65.36 ± 4.49^c^
ND-PO + PS 300	130.35 ± 2.80^c^	106.65 ± 2.82^b^	59.82 ± 1.85^b^

Each bar represents mean ± MSE; n = 5.

a p < 0.01.

b p < 0.001, significant difference compared to sham-operated control.

a p <0.05.

b p < 0.01.

c p < 0.001, significant difference compared to ND-PO control. **SHAM + H**_**2**_**O**: sham-operated rats receiving distilled water (10 mL/kg); **ND-PO + H**_**2**_**O**: ovariectomized rats with postmenopausal disorders and receiving distilled water (10 mL/kg); **ND-PO + E**_**2**_**V**: ovariectomized rats with postmenopausal disorders and receiving valerate estradiol (1 mg/kg); **ND-PO + PS 100, ND-PO + PS 200, ND-PO + PS 300**: ovariectomized rats with postmenopausal disorders and receiving *P. soyauxii* aqueous extract at 100, 200, and 300 mg/kg respectively.

#### 3.2.3 Anti-amnesic effects of PS

##### 3.2.3.1 Anti-amnesic effects of PS on short-term memory

**Fig. 3:**
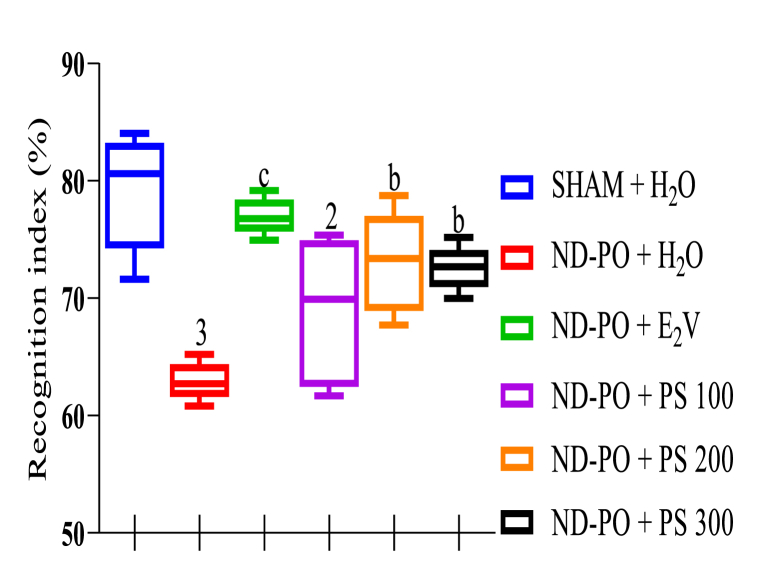
Determination of anti-amnesic potential of PS extract during NOR. Each bar represents mean ± MSE; n = 5; ^2^p < 0.01; ^3^p < 0.001, a significant difference compared to Sham control; ^b^p < 0.01; ^c^p < 0.001, a significant difference compared to ND-PO control. **SHAM + H**_**2**_**O**: sham-operated rats receiving distilled water (10 mL/kg); **ND-PO + H**_**2**_**O**: ovariectomized rats with postmenopausal disorders and receiving distilled water (10 mL/kg); **ND-PO + E**_**2**_**V**: ovariectomized rats with postmenopausal disorders and receiving valerate estradiol (1 mg/kg); **ND-PO + PS 100, ND-PO + PS 200, ND-PO + PS 300**: ovariectomized rats with postmenopausal disorders and receiving *P. soyauxii* aqueous extract at 100, 200, and 300 mg/kg respectively.

To assess the potential anti-amnesic impact of PS on short-term memory, the novel object recognition test (NOR) was carried out. As shown in Fig. 3, ovariectomized animals displayed a low recognition index (p < 0.01) compared to Sham-operated rats. Interestingly, treatment with PS extract at 200 and 300 mg/kg led to a significant increase (p < 0.01) in the recognition index compared to the ND-PO control group.

##### 3.2.3.2 Anti-amnesic effects of PS on long-term memory

**Fig. 4:**
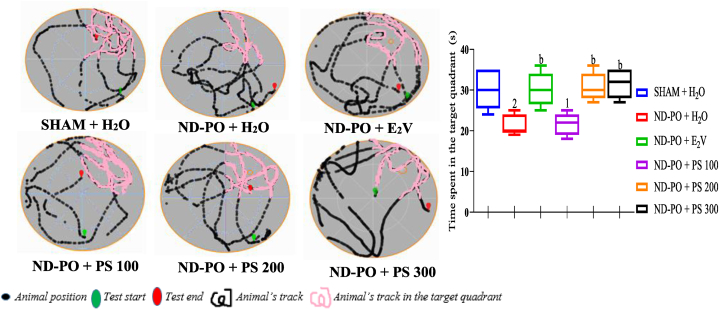
Determination of anti-amnesic potential of PS extract during MWM. Each bar represents mean ± MSE; n = 5; ^1^p < 0.05; ^2^p < 0.01, a significant difference compared to SHAM control; ^b^p < 0.01, a significant difference compared to ND-PO control. **SHAM + H**_**2**_**O**: sham-operated rats receiving distilled water (10 mL/kg); **ND-PO + H**_**2**_**O**: ovariectomized rats with postmenopausal disorders and receiving distilled water (10 mL/kg); **ND-PO + E**_**2**_**V**: ovariectomized rats with postmenopausal disorders and receiving valerate estradiol (1 mg/kg); **ND-PO + PS 100, ND-PO + PS 200, ND-PO + PS 300**: ovariectomized rats with postmenopausal disorders and receiving *P. soyauxii* aqueous extract at 100, 200, and 300 mg/kg respectively.

As depicted in Fig. 4, the time spent in the target dial was significantly reduced (p < 0.001) by ovariectomy in rats compared to sham control during the Morris water maze test (MWM). However, this time, oral ingestion of PS water extract at 200 and 300 mg/kg and estradiol valerate at 1 mg/kg significantly increased over (p < 0.01). Compared to ND-PO control, PS reduced the number by 30.80 ± 1.52 and 31.60 ± 1.60 at 200 and 300 mg/kg, respectively, and estradiol valerate decreased it by 30.20 ± 1.85 compared to ND-PO control animals that displayed 30.20 ± 2.17. Based on these findings, it can be concluded that treatment with PS and estradiol valerate significantly improved behavior disorders induced by chronic depletion of estrogen due to ovariectomy.

### 3.3 Antioxidant and neuromodulator effects of PS

Firstly, we examined the neuromodulation factors in the hippocampal homogenate of rats. As revealed by Table 3, rats with ovaries ablation displayed high activity of GABA-T (p˂0.01), a low level of total proteins (p˂0.01), calcium (p˂0.01), magnesium (p˂0.01), GABA (p˂0.01), 5-HT (p˂0.05), and ACh (p˂0.01) levels compared to sham control. In contrast, oral administration of PS at doses of 200 and estradiol valerate significantly reduced GABA-T activity (p˂0.01). Furthermore, the plant extract at the dose mentioned above significantly increased total proteins (p˂0.001), calcium (p˂0.05), magnesium (p˂0.01), GABA (p˂0.05), 5-HT (p˂0.05), and ACh (p˂0.01) levels of hippocampal homogenate compared to ND-PO rats.

**Table 3:** Effects of *P. soyauxii* on hippocampal biochemical and antioxidant parameters.

	SHAM + H_2_O	ND-PO + H_2_O	ND-PO + E_2_V	ND-PO + PS 100	ND-PO + PS 200	ND-PO + PS 300
GABA-T (μg/g/min/g x10^2^)	0.39 ± 0.02	0.61 ± 0.03^b^	0.43 ± 0.04^a^	0.41 ± 0.05	0.43 ± 0.02^a^	0.45 ± 0.02^a^
GABA (μg/g)	97.05 ± 5.29	76.47 ± 5.88^c^	91.17 ± 6.82^a^	77.94 ± 6.76^b^	92.64 ± 4.94^a^	88.17 ± 4.41
5-HT (μg/g)	65.33 ± 2.61	48.18 ± 3.31^c^	60.13 ± 3.45^a^	57.61 ± 2.40	60.66 ± 2.34^a^	49.26 ± 1.96
ACh (μg/g)	22.72 ± 1.56	15.52 ± 1.19^a^	25.81 ± 1.58^c^	20.42 ± 0.99	24.91 ± 2.38^b^	22.74 ± 0.96^a^
Ca^2+^ (mmoL/g)	1.62 ± 0.05	1.89 ± 0.02^b^	1.66 ± 0.04^b^	1.76 ± 0.06	1.69 ± 0.00^a^	1.75 ± 0.03
Mg^2+^ (mmoL/g)	0.74 ± 0.01	0.65 ± 0.00^b^	0.77 ± 0.01^c^	0.67 ± 0.01^a^	0.72 ± 0.01^b^	0.70 ± 0.00
MDA (mmol/gx10^1^)	2.04 ± 0.05	2.74 ± 0.09^b^	1.84 ± 0.09^c^	2.03 ± 0.09^b^	1.98 ± 0.21^b^	1.84 ± 0.06^c^
GSH (mmol/g)	0.45 ± 0.02	0.35 ± 0.01^c^	0.50 ± 0.01^c^	0.41 ± 0.03	0.49 ± 0.01^c^	0.44 ± 0.03^a^
Nitrites (mmol/g/x10^1^)	0.29 ± 0.02	0.18 ± 0.01^a^	0.29 ± 0.01^a^	0.31 ± 0.03^a^	0.33 ± 0.02^b^	0.29 ± 0.02^a^
Proteins	0.21 ± 0.00	0.16 ± 00^a^	0.26 ± 0.1^b^^,^^c^	0.20 ± 0.01	0.23 ± 0.01^c^	0.21 ± 0.00^b^

Values represent mean ± MSE; n = 5.

a p < 0.05.

b p < 0.01.

c p < 0.01, significant difference compared to Sham-operated control.

a p <0.05.

b p < 0.01.

c p < 0.001, significant difference compared to ND-PO control. **SHAM + H**_**2**_**O**: sham-operated rats receiving distilled water (10 mL/kg); **ND-PO + H**_**2**_**O**: ovariectomized rats with postmenopausal disorders and receiving distilled water (10 mL/kg); **ND-PO + E**_**2**_**V**: ovariectomized rats with postmenopausal disorders and receiving valerate estradiol (1 mg/kg); **ND-PO + PS 100, ND-PO + PS 200, ND-PO + PS 300**: ovariectomized rats with postmenopausal disorders and receiving *P. soyauxii* aqueous extract at 100, 200, and 300 mg/kg respectively.

Secondly, the levels of anti- and pro-oxidant factors were assessed. Table 3 illustrates that ovariectomy resulted in a significant decrease (p < 0.001) in the level of GSH in the hippocampal region compared to the Sham control. Treatment with the extract of PS at 200 and 300 mg/kg doses resulted in a significant increase (p < 0.001 and p < 0.05, respectively) in GSH levels compared to the ND-PO control. Furthermore, ND-PO rats presented lower nitrites in the hippocampal region than the Sham animals. Treatment with *P. soyauxii* extract at doses of 100, 200, and 300 mg/kg resulted in a significant increase in nitrite levels in ND-PO animals, respectively, of (p < 0.05), (p < 0.01), and (p < 0.05). Regarding MDA level, ovarian ablation led to its increase (p < 0.001), and the administration of PS at doses of 200 and 300 mg/kg resulted in a significant decrease (p < 0.01, p < 0.001, respectively) of this parameter.

### 3.4 Protective outcome of PS on hippocampus and neocortex

**Fig. 5:**
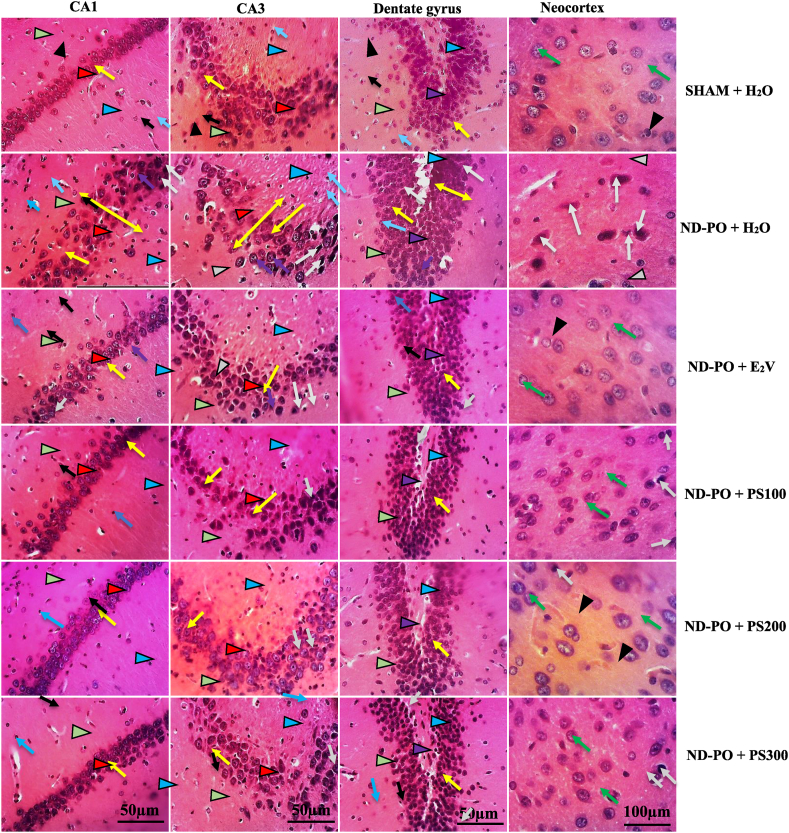
Representative hematoxylin-eosin staining of CA1, CA3, dentate gyrus (200x), and neocortex (400x) of parasagittal sections from controls and experimental groups. **SHAM + H**_**2**_**O**: Sham-operated rats displayed normal neurons in the polymorphic layer (**green arrowhead**), pyramidal cell layer (**red arrowhead**), and molecular layer (**blue arrowhead**) in both cornu Ammonis 1 and 3 (CA1 and CA3). In the dentate gyrus, nervous cells are organized into three layers, namely the polymorphic layer (**green arrowhead), the** granule cell layer (**purple arrowhead**), and the molecular layer (**blue arrowhead**). The polymorphic layer exhibits closely packed cell bodies of the normal pyramidal neurons (**yellow arrow**) that are regularly arranged in 3–4 rows and appear small with vesicular nuclei, prominent nucleoli, and scanty cytoplasm. The polymorphic layer and molecular layer display both deeply (**blue arrow**) and lightly (**black arrow**) stained nuclei of glial cells with normal blood capillaries (**black arrowhead**). The neocortical layer represents the external granular layer with packed cell bodies of normal non-pyramidal neurons (g**reen arrow**) with vesicular nuclei, prominent nucleoli, and scanty cytoplasm; **ND-PO + H**_**2**_**O**: ovariectomized control rats displayed hyperchromatic (**grey arrow**) and vacuolated (**purple arrow**) neurons in the pyramidal cell layer (**red arrowhead**) in both CA1 and CA3. The neurons of this layer are also disorganized (**double yellow arrow**). In the dentate gyrus, the granule cell layer (**purple arrowhead**) is disorganized and presents hyperchromatic (**grey arrow**) and vacuolated (**purple arrow**) neurons. The polymorphic layer and molecular layer display more deeply (**blue arrow**) than lightly (**black arrow**) stained nuclei of glial cells with dilated blood capillaries (**grey arrowhead**). The neocortical layer represents the external granular layer with packed cell bodies of hyperchromatic non-pyramidal neurons (**grey arrow**). **ND-PO + E**_**2**_**V**, **ND-PO + PS 100, ND-PO + PS 200, ND-PO + PS 300:** ovariectomized rats receiving estradiol valerate or PS extract displayed mild to moderate disorganization of the granule cell layer in CA1, CA3, and the dentate gyrus, with a low amount of hyperchromatic (**grey arrow**) and vacuolated (**purple arrow**) neurons in the pyramidal cell layer (**red arrowhead**) in both CA1 and CA3.

Histological analysis was performed to confirm the deleterious effects of heavy depletion of endogenous estrogen due to ovariectomy on the neuron, glial cells, and vascularization. According to Fig. 5, the H&E staining revealed that 112 days of estrogen depletion due to ovarian ablation led to an increase of hyperchromatic and vacuolated neurons in the pyramidal cell layer in both cornu Ammonis 1 and 3 (CA1 and CA3). The neurons of this layer are also disorganized. In the dentate gyrus, the granule cell layer is sloppy and presents hyperchromatic and vacuolated neurons. The polymorphic and molecular layers display more deeply than lightly stained nuclei of glial cells with dilated blood capillaries. The neocortical layer represents the external granular layer with packed cell bodies of hyperchromatic non-pyramidal neurons. Hippocampal neurons were dispersed in the CA1 region compared to the Sham control. Microscopic examination of the brain in PS/E_2_V-treated ND-PO rats revealed a significant decrease in hyperchromatic and vacuolated neurons in the hippocampal area and the cortex. PS-treated rats displayed mild to moderate disorganization of the granule cell layer in the CA1, CA3, and dendate gyrus.

### 3.5 Effects of PS on neuron counting in hippocampal areas

**Fig. 6:**
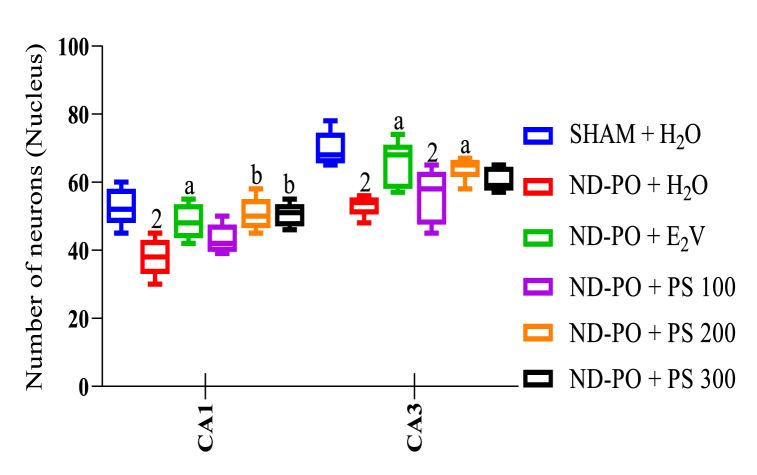
Effects of *P. soyauxii* on the number of neurons in hippocampal regions. Each bar represents mean ± MSE; n = 5; ^1^p < 0.05; ^2^p < 0.01; ^3^p < 0.001, significant difference compared to Sham-operated control; ^a^p <0.05; ^b^p < 0.01; ^c^p < 0.001, significant difference compared to ND-PO control. **SHAM + H**_**2**_**O**: sham-operated rats receiving distilled water (10 mL/kg); **ND-PO + H**_**2**_**O**: ovariectomized rats with postmenopausal disorders and receiving distilled water (10 mL/kg); **ND-PO + E**_**2**_**V**: ovariectomized rats with postmenopausal disorders and receiving valerate estradiol (1 mg/kg); **ND-PO + PS 100, ND-PO + PS 200, ND-PO + PS 300**: ovariectomized rats with postmenopausal disorders and receiving *P. soyauxii* aqueous extract at 100, 200, and 300 mg/kg respectively.

Fig. 6 presents the neurons counted in the CA1 and CA3 regions. The ovarian ablation induced a significant (p < 0.01) decrease in the number of neurons in the CA1 and CA3 regions. PS aqueous extract (200 mg/kg and 300 mg/kg) significantly (p < 0.01) increased the number of neurons in the CA1 hippocampal region compared to ND-PO animals. Similarly, in the CA3 region, *P. soyauxii* at 200 mg/kg, like estradiol valerate, significantly (p < 0.05) increased the number of neurons compared to ND-PO animals.

### 3.6 Principal component analysis

**Fig. 7:**
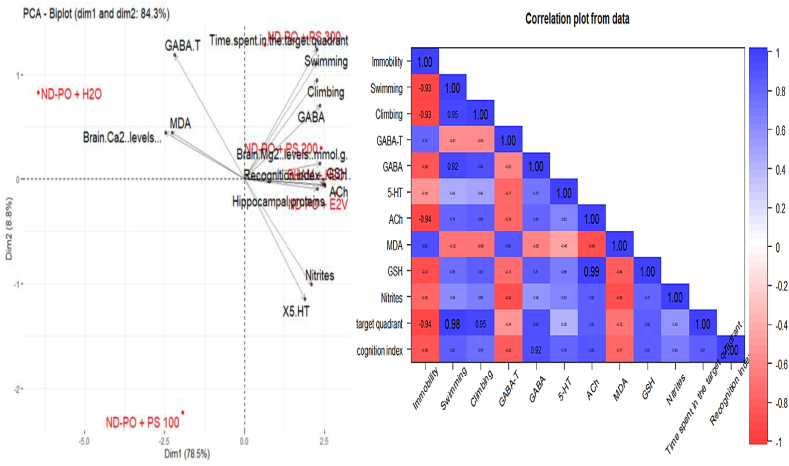
Correlation circle between the different variables and correlation matrix.

Principal component analysis (PCA) was used to classify these doses according to their activities (immobility, swimming, climbing, GABA-T, GABA, 5-HT, ACh, MDA, GSH, nitrites, time spent in the target quadrant, recognition index, hippocampal proteins, brain Ca^2+^ levels, and brain Mg^2+^ levels). Fig. 7 shows the correlation circle between the different variables. Accordingly, these variables contribute 84.3 % to forming the (F1*F2) system. The F1 axis alone explains 78.5 % of the variables observed, and the second axis defines 8.8 %.

### 3.7 Effects of PS on AChE and BChE activities

**Fig. 8:**
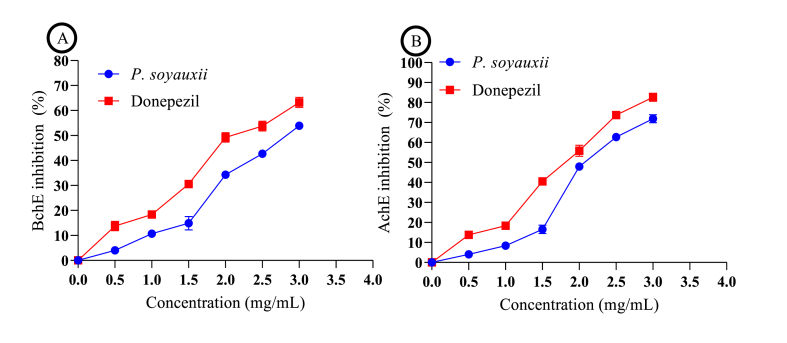
Effects of *P. soyauxii* on acetylcholinesterase and butyrylcholinesterase activities.

Various inhibition concentrations were determined using colorimetric assays to assess the potential of P. soyauxii to induce the inhibition of AChE and BChE activities. *P. soyauxii* exhibited significant inhibitory activity of AChE and BChE in a concentration-dependent manner, as shown in Fig. 8. This inhibition reached a maximum value of 53.89 ± 1.17 % at a concentration of 3 mg/mL with an IC_50_ of 2.66 ± 0.04 mg/mL for BChE activity (Figs. 8B) and 71.83 ± 1.23 % at a concentration of 3 mg/mL with an IC_50_ of 2.11 ± 0.08 mg/mL for AChE activity (Fig. 8A). Donepezil also significantly inhibited BChE activity in a concentration-dependent manner by 63.26 ± 1.82 % at the maximum concentration of 3 mg/mL, with an IC_50_ of 2.07 ± 0.03 mg/mL. The same applied to AChE, inhibited by 82.63 ± 3.02 % at the maximum concentration of 3 mg/mL, with an IC_50_ of 1.16 ± 0.06 mg/mL.

### 3.8 *In vitro* anti-inflammatory activity of PS

Based on the effects of PS on nitric oxide production, albumin denaturation, alkaline phosphatase, and 5-lipoxygenase activities, its anti-inflammatory potential was evaluated and reported in Table 4. *P. soyauxii* exhibited significant inhibitory activity on NO production and albumin denaturation in a concentration-dependent manner. This inhibition ranged from 23.28 ± 2.32 % to 87.94 ± 5.18 %, respectively, for concentrations of 0.1 μg/mL and 1000 μg/mL with an IC_50_ of 37.71 μg/mL for NO production and from 33.01 ± 0.12 % to 94.43 ± 2.23 %, respectively, for concentrations of 0.1 μg/mL and 1000 μg/mL with an IC_50_ of 85.71 μg/mL for albumin denaturation. In parallel, baicalin significantly inhibited NO production in a concentration-dependent manner, with a maximum effect of 97.43 % at 1000 μg/mL and an IC_50_ of 12.26 μg/mL, as well as albumin denaturation, with a maximum inhibition of 75.64 ± 0.70 % at 1000 μg/mL and an IC_50_ of 25.74 μg/mL. PS significantly increased alkaline phosphatase activity. This increase was concentration-dependent, ranging from 75.62 ± 2.79 % to 395.83 ± 15.35 % for concentrations of 0.1 μg/mL and 1000 μg/mL, respectively. These effects paralleled those of baicalin, which increased this activity from 150.39 ± 10.14 % to 769.26 ± 28.23 % for concentrations of 0.1 μg/mL and 1000 μg/mL, respectively. PS exhibited significant inhibitory activity on 5-lipooxygenase activity in a concentration-dependent manner. This inhibition was maximal at 1000 μg/mL and 68.43 ± 2.52 %, with an IC_50_ of 92.74 μg/mL. Baicalin decreased this activity in a concentration-dependent manner, with a maximum inhibition of 94.29 ± 1.37 % at 1000 μg/mL and an IC_50_ of 22.85 μg/mL.

**Table 4:** *In vitro* determination of anti-inflammatory effects of PS.

Concentration (μg/mL)		1000	100	10	1	0.1	IC_50_ (μg/mL)
Inhibition of NO production (%)	***P. soyauxii***	87.94 ± 5.18	73.90 ± 2.37	42.21 ± 9.01	34.24 ± 3.17	23.28 ± 2.32	37.71
	**Baicalin**	94.43 ± 2.23	89.17 ± 1.09	49. ± 2.25	38.59 ± 3.54	33.01 ± 0.12	12.26
Inhibition of albumin denaturation (%)	***P. soyauxii***	63.64 ± 1.26	52.14 ± 0.80	43.11 ± 3.18	29.46 ± 1.94	17.44 ± 0.88	85.71
	**Baicalin**	75.64 ± 0.70	58.47 ± 1.44	46.78 ± 1.18	34.46 ± 0.60	22.22 ± 0.92	25.74
ALP activity (%)	***P. soyauxii***	395.83 ± 15.35	215.49 ± 18.48	181.62 ± 11.51	155.25 ± 3.45	75.62 ± 2.79	/
	**Baicalin**	769.26 ± 28.23	229.56 ± 15.21	199.62 ± 9.25	177.51 ± 7.23	150.39 ± 10.14	/
Inhibition of 5-lipoxygenase activity (%)	***P. soyauxii***	68.43 ± 2.52	53.73 ± 4.57	30.82 ± 3.82	28.47 ± 2.56	12.58 ± 1.64	92.74
	**Baicalin**	94.29 ± 1.37	85.15 ± 1.23	52.30 ± 3.96	40.88 ± 1.21	23.75 ± 1.58	22.85

Each value represents the percentage of inhibition and is expressed as mean ± SEM; n = 3.

### 3.9 *In vitro* antioxidant potential of PS

The total antioxidant capacity and activity of PS on lipid peroxidation and NADPH-o inhibition were determined and shown in Table 5. The results show a concentration-dependent total antioxidant capacity of *P. soyauxii* with a maximum value of 43.08 ± 1.07 μg EE of ascorbic acid/g MS at 1500 μg/mL. It exhibited significant concentration-dependent inhibitory activity on NADPH-o activity and lipid peroxidation. This inhibition reached a maximum value of 67.77 ± 2.17 % at a concentration of 1500 μg/mL with an IC_50_ of 524.25 ± 12.54 μg/mL for NADPH-o activity and 61.37 ± 1.11 % at a concentration of 1500 μg/mL with an IC_50_ of 684.27 ± 8.47 μg/mL for lipid peroxidation. Vitamin C also significantly inhibited NADPH-o activity in a concentration-dependent manner by 74.62 ± 1.42 % at the maximum concentration of 1500 μg/mL, with an IC_50_ of 382.12 ± 7.23 μg/mL. The same applied to lipid peroxidation, which was inhibited by 79.22 ± 2.52 % at the maximum concentration of 1500 μg/mL, with an IC_50_ of 386.52 ± 5.45 μg/mL.

**Table 5:** *In vitro* determination of the antioxidant potential of PS.

Concentration (μg/mL)	TAC (μg EE of ascorbic acid/g MS)	Inhibition of NADPH-o activity (%)		Inhibition of lipid peroxidation (%)	
		*P. soyauxii*	Vit C	*P. soyauxii*	Vit C
1500	43.08 ± 1.07	67.77 ± 2.17	74.62 ± 1.42	61.37 ± 1.11	79.22 ± 2.52
750	36.89 ± 0.97	57.06 ± 2.09	63.49 ± 5.31	52.11 ± 1.19	67.21 ± 2.60
375	19.79 ± 0.57	33.48 ± 3.36	49.63 ± 4.25	32.22 ± 2.12	51.23 ± 1.25
187.5	10.67 ± 0.23	19.14 ± 3.63	31.63 ± 3.58	18.10 ± 2.43	33.23 ± 2.11
93.75	8.77 ± 0.14	8.58 ± 3.53	18.75 ± 0.60	11.51 ± 1.23	24.55 ± 1.60
IC_50_ (μg/mL)	/	524.25 ± 12.54	382.12 ± 7.23	684.27 ± 8.47	386.52 ± 5.45

Each value represents the percentage of inhibition and is expressed as mean ± SEM; n = 3.

**TAC:** Total antioxidant capacity.

### 3.10 *In silico* profiling of AChE, MOA A, and GABA-T inhibition

**Fig. 9:**
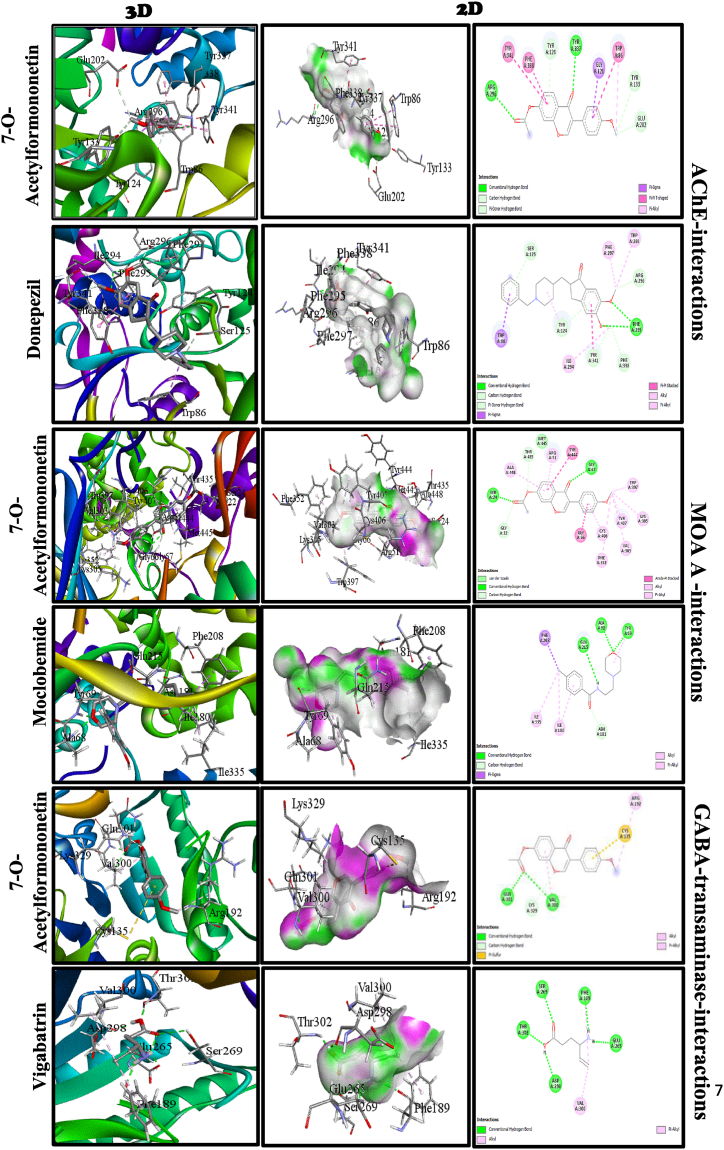
2D and 3D views of the molecular interactions between the amino acid residues of AChE, MAO-A, and GABA aminotransferase and 7-*O*-acetylformonetin and donepezil, moclobemide, and vigabatrin.

Table 6 shows the results of predicting compounds identified as crossing the BBB. This table shows that one identified molecule, 7-*O*-Acetylformononetin, can cross the blood-brain barrier. To verify the binding ability between key enzymes, molecular docking was performed using AutoDock 4.2, and the results were visualized using the Discovery Studio Visualizer. The critical affinity results of the 7-*O*-acetyl formononetin, an isoflavone contained in PS aqueous extract, against the selected AChE, MOA A, and GABA aminotransferase are shown in Table 7. The docking score of the identified *Pterocarpus soyauxii* compound capable of crossing the BBB is −8.71 kcal/mol, −9.69 kcal/mol, and −8.13 kcal/mol, respectively, for AChE, MOA A, and GABA. The 7-*O*-acetyl formononetin crosses the BBB and reacts with residues of the acyl pocket, the anionic site, and the peripheral site of AChE, such as Donepezil (reference inhibitor). In addition, this PS isoflavone interacts with Gly 121 of the Oxyanion hole of AChE to the detriment of Donepezil. The 7-*O*- acetylformononetin also reacts with Lys 305, Trp 397, Tyr 407, and Tyr 444 on the active site of the MOA A. On the active site of the GABA-T, the target isoflavone reacts with Arg 192, while Vigabatrin interacts with Glu 265 (Fig. 9).

**Table 6:** Prediction of BBB crossing by *Pterocarpus soyauxii* compounds and Donepezil.

Compounds	3′,5′-dimethoxy-4-stilbenol	7-*O*-Acetylformononetin	Cembrene	Khrinone A	Donepezil
BBB Penetration	**-**	**+**	**-**	**-**	**+**

**Table 7:** Molecular docking scores of the compound against AChE, MAO-A, and GABA-T and profiles of their essential amino acid residues that interact with the identified ligand.

Name of the Compound	Binding energy (Kcal/mol)	Inhibition constant (kI) (μM)	Inter molecular Energy (Kcal/mol)	Catalytic triad	Oxyanion hole	Acyl pocket	Anionic site
AChE							
7-*O*-Afn	−8.71	0.414	−9.9	/	Gly121	Phe338	Trp86
Donepezil	−10.91	0.01 −12.7	/	/	Phe295, Phe297, Phe338	Trp86	−10.91
MAO A							
7-*O*-Afn	−9.69	0.078	−10.89	Lys 305, Trp 397, Tyr 407 and Tyr 444	/	/	/
Moclomebide	−7.66	2.41	−8.86	/	/	/	/
GABA-T							
7-*O*-Afn	−8.13	1.1	−9.32	Arg 192	/	/	/
Vigabatrin	−4.43	566.16	−6.22	Glu 265	/	/	/

7-*O*-Afn: 7-*O*-acetylformononetin

## 4 Discussion

The current research focuses on the prevalence of neurological conditions associated with menopause. Low estrogen levels enhance the emergence of neuronal impairment in postmenopausal women, although neuronal death rises with age [53]. The number of patients with neurological disorders associated with postmenopause (ND-PO) is increasing rapidly owing to the rate of the global aging trend, particularly in women. As a result, there is a growing demand for non-hormonal treatments for the relief of menopause-linked disorders [3]. The use of medicinal plants and their derivatives as a rich source of therapeutic compounds and structural variety has been acknowledged for a long time. Concerning *Pterocarpus soyauxii* (PS) aqueous extract, the current study set out to 1) examine its phytochemical composition, 2) evaluate PS aqueous extract's neuroprotective effects in rats, and 3) investigate probable underlying mechanisms. In rats, long-term ovariectomy has been shown to cause neuropsychic problems such as depression, anxiety, and memory loss [20,54,55]. The findings of the present study demonstrate that 16-week ovariectomy caused anxiety, as shown by a more significant delay in biting food in an unpleasant environment during the NSF, depression reflected in a lower time spent swimming and climbing, and higher immobility during the FS. Amnesia was also noted, marked by decreased time spent in the target frame during the MWM and in the recognition index in the NOR. These results are similar to those of Djiogue et al. [7], Ngoungoure et al. [16], and Zhou et al. [55], who showed the same variation in behavioral parameters assessed during the tests as mentioned earlier in ovariectomized rodents.

Post-oophorectomy estrogen deficiency is likely to lead to neuropsychological disorders via a variety of mechanisms. Firstly, inducing oxidative stress through increased NADPH-oxidase (NADPH-o) activity [10]. This oxidative stress has long been cited as playing a significant role in the pathogenesis of nervous disorders, particularly memory loss, anxiety, and depression [56], and secondly, by inducing brain inflammation via activation of microglial cells [9]. In fact, reactive oxygen species derived from NADPH-o in nerve cells have also been implicated in the progression of central nervous system diseases by inducing myelin phagocytosis and damage to the myelin sheath [57]. In this study, cerebral oxidative stress was marked by increased MDA levels and decreased GSH levels in the hippocampal areas. Principal component analyses showed a positive correlation between MDA and GSH levels and the behavioral parameters assessed, reflecting the onset of anxiety, depression, and memory disorders, suggesting that oxidative stress was involved in the onset of neuropsychological disorders in this study. The aqueous extract of *P. soyauxii* protected against the nervous disorders observed in rats by reversing the behavioral parameters assessed at the end of each behavioral test. The current study showed that PS antioxidant activity mediates the anxiolytic, antidepressant, and anti-amnesic effects. Previous studies have shown that the aqueous extract of *Pterocarpus soyauxii* exhibits antioxidant activity *in vitro* on DPPH and ABTS radicals. This suggests that *P. soyauxii* can stabilize free radicals by donating an electron or capturing a proton [19]. In addition to these stabilizing mechanisms, our results show that *P. soyauxii in vitro* inhibits the activity of NADPH-o, the main oxidative enzyme in cellular mitochondria [58,59], and inhibits lipid peroxidation. This mechanism generates MDA and cell death by necrosis [60]. These *in vitro* mechanisms explain the drop in MDA and rise in GSH levels in hippocampal homogenates and attest to the antioxidant power of *P. soyauxii*. Molecular modeling confirms that the interaction of certain polyphenols, classes of molecules identified and quantified in *P. soyauxii* extract, can interact with the flavin adenine dinucleotide (FAD) binding pockets of NADPH-o via hydrogen bonds and hydrophobic interactions to inhibit the oxidative activity of NADPH-o thus preventing oxidative neuronal damage [61].

In addition, inhibition of lipid peroxidation would explain *P. soyauxii's* protection against the fall in hippocampal neuron numbers in the present work. Indeed, according to several authors, MDA is used as a marker of cell dysfunction or death [62]. According to the quantitative phytochemical analysis, these neuroprotective effects linked to the antioxidant power of *P. soyauxii* can be attributed to the flavonoids and polyphenols found in the plant extract. Polyphenols are known to be modulators of NADPH-o activity [62], thus preventing cell death due to oxidative damage. The present study showed that PS contains considerable levels of polyphenols. Besides, considering that the final phytoestrogen concentration in general, and the isoflavone concentration in particular, is influenced by the post-harvest processing, this study reveals the average levels of target phytoestrogens contained in PS with pharmacological activities on brain disorders due to menopause. The scavenging potential of PS corroborates with the prevention of the decline in the number of neurons in the CA1 and CA3 regions of the hippocampus observed in ovariectomized rats. Hippocampal neuronal death has been reported in both ovariectomized rats and postmenopausal women [63]. The oxidative stress observed in the brain during menopause or in rodent models of menopause, which is often linked to anxiety and depression, is also thought to result from excitotoxicity following the activation of NMDA receptors by glutamate [64]. Excitotoxicity is related to intracellular calcium overload, which, by activating NADPH-oxidase, generates the formation of ROS and induces oxidative neuronal damage and neuronal loss [[65], [66], [67]]. This pathway is partially verified in this work by the increase in brain homogenates of Ca^2+^ ions and the decrease in Mg^2+^ in the functioning of NMDA channel receptors following their activation. Ca^2+^ ion influx and Mg^2+^ ion efflux in the brain are often strongly correlated with anxiety and depression [64]. *P. soyauxii* extract's reduction in Ca^2+^ and increase in Mg^2+^ in the brain could contribute to the anxiolytic and antidepressant properties of the plant extract. Genistein, a well-studied phytoestrogen, is reported to induce such modulation on NMDA receptors [68]. By the way, *P. soyauxii,* given its established estrogenic activity [19], could probably possess a similar potential.

Inflammation is now considered a significant factor in the pathogenesis of nervous disorders such as anxiety, depression, and memory disorders [9,[69], [70], [71]]. There is a high incidence of neuroinflammation in women with advancing age in postmenopausal women [72]. This inflammation is thought to be mediated by several pathways, including lipoxygenases and iNOS [73,74]. Interestingly, the expression of 5-lipoxygenase and its metabolites increases with age, both in animal models and in human subjects. 5-lipoxygenase protein levels are exceptionally high in the cortex and hippocampus, areas known to be particularly vulnerable to neurodegeneration and Alzheimer's disease in particular, as well as depression [75,76]. 5-lipoxygenase promotes lipid peroxidation *in vitro* and *in vivo* [77,78]. Leukotrienes, the metabolic products of 5-lipoxygenase activation, trigger chemotaxis by immune cells and are critical molecular players in CNS inflammatory pathophysiology [79]. Similarly, the inducible nitric oxide synthase (iNOS), which is expressed under the action of inflammatory agents in brain cell overstimulation, leads to the uncoupling of NOS dimers, resulting in massive peroxynitrite production [80,81]. As a result, peroxynitrite nitration of free and protein-bound tyrosine residues forms nitrotyrosine, leading to structural disruption and protein dysfunction [82,83]. A previous study showed that peroxynitrite leads to protein nitration and is highly toxic to neurons, inducing impaired synaptic transmission [84]. In this study, *in vitro* data revealed that the aqueous extract of *P. soyauxii* heartwood inhibits NO production, increases alkaline phosphatase activity, and inhibits 5-lipoxygenase and protein denaturation. These effects suggest that the aqueous extract of *P. soyauxii* has anti-inflammatory activity and is a candidate for treating menopause-related nervous disorders. Indeed, previous work has shown that certain flavonoid phytoestrogens quantified in this study can inhibit NO production by inhibiting iNOS and 5-lipoxygenase activity [85]. Furthermore, the pterostilbene identified by LCMS analysis following the earlier work of Mengue et al. [19] in the aqueous extract of the heartwood of *Pterocarpus soyauxii* have anti-inflammatory activities through inhibition of NF-κB phosphorylation [86]. Nevertheless, there is a need to investigate the effects of *P soyauxii* on neuroinflammation *in vivo*.

In addition to oxidative stress and inflammation as pathogenic factors in postmenopausal memory loss, anxiety, and depression, the disruption of GABAergic, serotonergic, and cholinergic neurotransmission is no less critical. Oophorectomy or menopause is associated with a drop in 5-HT, GABA, and ACh levels in the cerebral cortex, hypothalamus, amygdala, and especially the hippocampus [[87], [88], [89], [90]]. According to several authors, these alterations are a plausible etiology that would support the pathogenesis of memory, depression, and anxiety disorders in conditions of estrogen deficiency [91]. Nelson et al. [63] have shown that anxiety, memory disorders, and depression are linked to reduced concentrations of monoamines (5-HT), ACh, and GABA in the hippocampus of ovariectomized rats. Our results agree with these observations, in which neurobehavioral disorders in ovariectomized rats were positively correlated with decreases in the levels of the neuromodulators, as mentioned earlier. Pathophysiological mechanisms explaining this drop in neurotransmitter levels include post-ovariectomy or postmenopausal cellular oxidative damage responsible for the death of GABAergic, serotonergic, and cholinergic neurons [92] and increased activity of enzymes degrading these neuromodulators (GABA-T, MOA, and AChE). This work's increase in GABA-T activity in ovariectomized rat brain homogenates aligns with these pathways. *P. soyauxii* corrected the fall in these neurotransmitters, suggesting that *P. soyauxii* may have chemical compounds capable of modulating the activity of neurotransmitter-degrading enzymes or limiting the death of neurons responsible for their release. The results show that *P. soyauxii* corrected the drop in 5-HT, GABA, and ACh levels. These effects are linked to its ability to inhibit AChE and increase ACh release, as shown *in vitro* in this work and the work of Ganiyu et al. [93] and *in vivo* in that of Saliu et al. [16]. The increase in ACh levels by *P. soyauxii* would explain its anti-amnesic properties, with ACh being the main neuromediator responsible for memory [94]. This inhibition was confirmed *in silico* by the ability of 7-*O*-acetylformonetin, an isoflavone contained in *P. soyauxii* extract, according to the LC-MS analysis performed by Mengue et al. [19], to bind to the functional sites of AChE. 7-*O*-acetylformonetin can induce the inhibition of AChE with an appreciable inhibition constant. Isoflavone can contribute to minimizing the impact of memory impairment in post-menopausal women [95]. Similarly, the antidepressant and anxiolytic activity of *P. soyauxii* is thought to be mediated by the inhibition of monoamine oxidase and GABA-T. This was demonstrated *in silico* by the ability of 7-*O*-acetylformonetin to bind with high affinity to the catalytic sites of these enzymes and effectively inhibit them, as shown by the respective inhibition constants for each enzyme. *In vivo*, this inhibition would explain the increase in 5-HT and GABA levels in ovariectomized rats after *P. soyauxii* treatment. This study shows that the extract of PS is rich in polyphenolic, flavonoids, and tannin compounds and possesses potent antioxidant and anti-inflammatory activities. The *in-silico* model presented excellent predictions of the relative inhibitory effect of 7-*O*-acetylformonetin, an isoflavone contained in *P. soyauxii*, as its modulation of cholinergic, GABAergic, and monoaminergic systems. Further studies with MD simulation of 100ns followed by free energy calculations are needed to confirm the theoretical results on the affinity of 7-*O*-acetylformonetin. The neuroprotective activities of PS extract were confirmed in an ovariectomized-induced post-menopausal model. Further studies are warranted to investigate the fingerprint profile of organic extracts of PS and, further, the mechanisms involved in cholinergic, GABAergic, and monoaminergic modulation by PS and their potential application for the treatment of ND-PO.

## 5 Conclusions

According to our research, *P. soyauxii* aqueous extract could protect against nervous disorders due to low estrogen levels. Indeed, the extract contains high levels of polyphenols and flavonoids with antioxidant and anti-inflammatory effects. Additionally, the African padauk can modulate the neurotransmission of GABAergic, cholinergic, and serotoninergic systems. *In silico* molecular docking confirmed the neurochemical modulation, which showed that the 7-*O*-acetyl formononetin can cross the BBB and modulate these neuronal systems. This study then demonstrates the potential neuroprotective effects of *P. soyauxii*. The findings of this study suggest that *P. soyauxii* could be beneficial for improving cognitive parameters in estrogen-deficient states and contribute to maintaining excellent psychological health in postmenopausal women. Therefore, the current study can justify using this plant in traditional folk medicine to alleviate menopause-related disorders.

## Ethics approval and consent to participate

Not applicable.

## Funding

This research did not receive funding from public, commercial, or not-for-profit sectors.

**Table:** Abbreviation

5-HT	:	Serotonin
ACh	:	Acetylcholine
GD	:	Dentate gyrus
CA1	:	Cornu Ammonis 1
CA3	:	Cornu Ammonis 3
GABA	:	Gamma-aminobutyric acid
GABA-T	:	GABA-transaminase
GSH	:	Reduced glutathione
HRT	:	Hormone replacement therapy
MDA	:	Malondialdehyde
MNDA	:	N-methyl-d-aspartate
*PS*	:	*Pterocarpus soyauxii*

## Availability of data and material

The authors have confirmed that the data supporting the findings of this study are available in the article.

## CRediT authorship contribution statement

**Pascal Emmanuel Owona:** Writing – original draft, Methodology, Investigation, Formal analysis, Data curation, Conceptualization. **Yolande Sandrine Mengue Ngadena:** Writing – review & editing, Writing – original draft, Validation, Supervision, Resources, Methodology, Formal analysis, Conceptualization. **Danielle Claude Bilanda:** Writing – review & editing, Supervision, Resources, Project administration. **Madeleine Chantal Ngoungouré:** Writing – original draft, Methodology, Investigation. **Lohik Mbolang Nguegan:** Methodology, Investigation, Data curation. **Ronald Bidingha A Goufani:** Methodology, Investigation, Data curation. **Rivaldo Bernes Kahou Tadah:** Methodology, Investigation, Data curation. **Michel Noubom:** Methodology, Investigation, Formal analysis, Data curation. **Armand Fils Ella:** Methodology, Investigation, Formal analysis, Data curation. **Yannick Carlos Tcheutchoua:** Methodology, Investigation, Formal analysis, Data curation. **Bruno Dupon Ambamba Akamba:** Methodology, Investigation, Formal analysis. **Paule Cynthia Bouguem Yandja:** Methodology, Investigation, Formal analysis, Data curation. **Paulin Keumedjio Teko:** Methodology, Investigation. **Paul Desire Dzeufiet Djomeni:** Writing – review & editing, Validation, Supervision, Conceptualization.

## Declaration of competing interest

The authors declare that they have no known competing financial interests or personal relationships that could have appeared to influence the work reported in this paper.

## References

[1] Crandall C.J., Mehta J.M., Manson J.E. (2023). Management of menopausal symptoms: a Review. JAMA.

[2] Soares C.N. (2019). Depression and menopause: an update on current knowledge and clinical management for this critical window. Med. Clin..

[3] United Nations (2019). Department of Economic and social affairs, population division. World Population Ageing 2019: Highlights (ST/ESA/SER.A/430).

[4] Heidari M., Ghodusi M., Rafiei H. (2017). Sexual self-concept and its relationship to depression, stress and anxiety in postmenopausal Women. J Menopausal Med.

[5] Alzheimer’s Association (2017). Alzheimer's disease facts and figures. Alzheimers Dement.

[6] Sales S., Ureshino R.P., Pereira R.T., Luna M.S., Pires de Oliveira M., Yamanouye N., Godinho R.O., Smaili S.S., Porto C.S., Abdalla F.M. (2010). Effects of 17 beta-estradiol replacement on the apoptotic effects caused by ovariectomy in the rat hippocampus. Life Sci..

[7] Djiogue S., Djiyou A.B., Seke P.F., Ketcha G.J.M., Djikem R.N., Njamen D. (2018). Memory and exploratory behavior impairment in ovariectomized Wistar rats. BBF.

[8] Michael C. (2016). Why we need research about autism and ageing. Autism: the international journal of research and practice.

[9] Fei G., Yang H., Lu W., Shi H., Chen Q., Luo Y., Liu L., Yan J. (2020). Ovariectomy induces microglial cell activation and inflammatory response in rat prefrontal cortices to accelerate the chronic unpredictable stress-mediated anxiety and depression. BioMed Res. Int..

[10] Farajdokht F., Sadigh-Eteghad S., Vatandoust S., Hosseini L., Morsali S., Feizi H., Mahmoudi P.G. Shadbad J. (2024). Sericin improves memory impairment via activation of the PKA-CREB-BDNF signaling pathway and suppression of oxidative stress in ovariectomized Mice. Neurochem. Res..

[11] Alyson A., Drummond G.R., Mast A.E., Schmidt H.H., Sobey C.G. (2007). Effect of gender on NADPH-oxidase activity, expression, and function in the cerebral circulation: role of estrogen. Stroke.

[12] Facecchia K., Fochesato L.A., Ray S.D., Stohs S.J., Pandey S. (2011). Oxidative toxicity in neurodegenerative diseases: role of mitochondrial dysfunction and therapeutic strategies. J. Toxicol..

[13] Resnick S.M., Maki P.M., Rapp S.R., Espeland M.A., Brunner R., Coker L.H., Granek I.A., Hogan P., Ockene J.K., Shumaker S.A. (2006). Women's Health Initiative Study of cognitive aging investigators, Effects of combination estrogen plus progestin hormone treatment on cognition and affect. J. Clin. Endocrinol. Metab..

[14] Chen W.Y., Hankinson S.E., Schnitt S.J., Rosner B.A., Holmes M.D., Colditz G.A. (2004). Association of hormone replacement therapy to estrogen and progesterone receptor status in invasive breast carcinoma. Cancer.

[15] Dalal P.K., Agarwal M. (2015). Postmenopausal syndrome. Indian J. Psychiatry.

[16] Ngoungoure M.C., Dzeufiet D.P.D., Bilanda D.C., Mengue N.Y.S., Mbolang N.L., Mballa M.F., Kameni P.M., Kamtchouing P. (2019). Neuroprotective effects of the *Anthocleista Schweinfurthii* Gilg. (Loganiaceae) stem bark extract in postmenopause-like model of ovariectomized wistar rats. J. Compl. Integr. Med..

[17] Saliu J.A., Oboh G., Omojokun O.S., Rocha J.B.T., Schetinger M.R.J., Guterries J., Stefanello N., Carvalho F., Schmatz R., Morsch V.M., Boligon A. (2016). Effect of dietary supplementation of Padauk (*Pterocarpus soyauxii*) leaf on high fat diet/streptozotocin induced diabetes in rats' brain and platelets. Biomed. Pharmacother..

[18] Saslis-Lagoudakis C.H., Klitgaard B.B., Forest F., Francis L., Savolainen V., Williamson E., Hawkins J.A. (2011). The use of phylogeny to interpret cross-cultural patterns in plant use and guide medicinal plant discovery: an example from Pterocarpus (Leguminosae). PLoS One.

[19] Mengue N.Y.S., Owona P.E., Noubom M., Mbock M.A., Mbolang N.L., Ngoungouré M.C., Fifen R.N., Bidingha A.C.R., Kahou T.R.B., Bilanda D.C., Kamtchouing P., Dzeufiet D.P.D. (2021). Estrogenic and antioxidant activities of *Pterocarpus soyauxii* (Fabaceae) heartwood aqueous extract in bilateral oophorectomized Wistar rat. eCAM.

[20] Mengue N.Y.S., Owona P.E., Bilanda D.C., Bidingha A.C.R., Tcheutchoua Y.C., Dzeufiet D.P.D. (2023). Postmenopausal status exacerbates stress-related neurological disorders in Rats. J. Adv. Biol. Biotechnol..

[21] Zhishen J., Mengcheng T., Jianming W. (1999). The Determination of flavonoid contents in mulberry and their scavenging effects on superoxide radicals. Food Chem..

[22] Broadhurst R.B., Jones T.W. (1978). Analysis of condensed tannins using acidified vanillin. J. Sci. Food Agric..

[23] Li H., Li S.L., Wu Z.W., Gong L., Wang J.L., Li Y.Z. (2008). Effect of traditional Chinese herbal Bu-Wang-San on synaptic plasticity in ovariectomised rats. J. Pharm. Pharmacol..

[24] Alena L., Mingming Z., Nathalie C., Mark S., Joshua A., Jasmine H., Maria B., Josko L., Mark D. (2003). Altered depression-related behaviors and functional changes in the dorsal raphe nucleus of serotonin transporter-deficient mice. Biol. Psychiatr..

[25] Erika E., Alonso F., Carolina L. (2003). Antidepressant-like effect of different estrogenic compounds in the Forced Swimming Test. Neuropsychopharmacol.

[26] Andrzej A., Kołat E., Różyk-Myrta A. (2014). In search of memory tests equivalent for experiments on animals and humans. Med Sci Monit.

[27] Morris R. (1984). Developments of a water-maze procedure for studying spatial learning in the rat. J. Neurosci. Methods.

[28] Clarck W.S., Balinski S., Marie A. (1975). Spectrometric study of a direct determination of serum calcium. Microchem. J..

[29] Treiz N.W. (1983).

[30] Nayak P., Chatterjee A.K. (2001). Effects of aluminium exposure on brain glutamate and GABA systems: an experimental study in rats. FCT.

[31] Yoshitake T., Kehr J., Yoshitake S., Fujino K., Nohta H., Yamaguchi M. (2004). Determination of serotonin, noradrenaline, dopamine and their metabolites in rat brain extracts and microdialysis samples by column liquid chromatography with fluorescence detection following derivatization with benzylamine and 1,2-diphenylethylenediamine. Chromatogr B Analyt Technol Biomed Life Sci..

[32] Hestrin S. (1949). The reaction of acetylcholine and other carboxylic acid derivatives with hydroxylamine, and its analytical application. JBC.

[33] Wilbur K.M., Bernheim F., Shapiro O.W. (1949). The thiobarbituric acid reagent as a test for the oxidation of unsaturated fatty acids by various agents. Arch. Biochem. Biophys..

[34] Ellman G.L. (1959). Tissue sulfhydryl groups. Arch. Biochem. Biophys..

[35] Green L.C., Wagner D.A., Glogowski J., Skipper P.L., Wishnok J.S., Tannenbaum S.R. (1982). Analysis of nitrate, nitrite, and [15N]nitrate in biological fluids. Anal. Biochem..

[36] Prieto P., Pineda M., Aguilar M. (1999). Spectrophotometric quantitation of antioxidant capacity through the formation of a phosphomolybdenum complex: specific application to the determination of vitamin E. Anal. Biochem..

[37] Moukette B., Pieme C.A., Nya P.C., Ngogang J.Y. (2015). *In vitro* antioxidant and anti-lipoperoxidative activities of bark extracts of *Xylopia aethiopica* against ion-mediated toxicity on liver homogenates. J. Complement. Integr. Med..

[38] Manosroi A., Saraphanchotiwitthaya A., Manosroi J. (2003). Immunomodulatory activities of *Clausena excavata* Burm. f. wood extracts. J. Ethnopharmacol..

[39] Titus R.G., Gueiros-Filho F.J., de Freitas L.A., Beverley S.M. (1995). Development of a safe live Leishmania vaccine line by gene replacement. Proc. Natl. Acad. Sci. U.S.A..

[40] Bansal S.K. (1987). Carbohydrate metabolism in the rat peritoneal macrophages. J Biosci.

[41] Sun J., Zhang X., Broderick M., Fein H. (2003). Measurement of Nitric Oxide Production in biological systems by using Griess reaction assay. Sensors.

[42] Suzuki Y., Orellana M.A., Schreiber R.D., Remington J.S. (1988). Interferon-gamma: the major mediator of resistance against *Toxoplasma gondii*. Science.

[43] Elias G., Rao M.N. (1988). Inhibition of albumin denaturation and anti-inflammatory activity of dehydrozingerone and its analogs. IJEB.

[44] Yougbare-Ziebrou M.N., Ouedraogo N., Lompo M., Bationo H., Yaro B., Gnoula C., Sawadogo W.R., Guissou I.P. (2016). Activités anti-inflammatoire, analgésiques et antioxydante de l’extrait aqueux des tiges feuillées de Saba senegalensis Pichon (Apocynaceae). Phytothérapie.

[45] Kostelnik A., Pohanka M. (2018). Inhibition of acetylcholinesterase and butyrylcholinesterase by a plant secondary metabolite Boldine. BioMed Res. Int..

[46] Kuppusamy A., Arumugam M., George S. (2017). Combining *in silico* and *in vitro* approaches to evaluate the acetylcholinesterase inhibitory profile of some commercially available flavonoids in the management of Alzheimer's disease. Int. J. Biol. Macromol..

[47] Daina A., Michielin O., Zoete V. (2017). SwissADME: a free web tool to evaluate pharmacokinetics, drug-likeness and medicinal chemistry friendliness of small molecules. Sci. Rep..

[48] Zhu H., Martin T.M., Ye L., Sedykh A., Young D.M., Tropsha A. (2009). Quantitative structure-activity relationship modeling of rat acute toxicity by oral exposure. Chem. Res. Toxicol..

[49] Chahad A.M., Adey S.A., Chatté A., Louis Z., Dijoux-Franca M.G. (2021). Chemical analysis and biological properties of *Terminalia macroptera* guill. & perr. From eastern Chad. Mediterr. J. Chem.

[50] Madeswaran A., Umamaheswari M., Asokkumar K., Sivashanmugam T., Subhadradevi V., Jagannath P. (2012). Computational drug discovery of potential phosphodiesterase inhibitors using studies 2012. Bangladesh J. Pharmacol..

[51] Goodsell D.S., Morris G.M., Olson A.J. (1996). Automated docking of flexible ligands: applications of AutoDock. J. Mol. Recognit.

[52] Nan X., Sun Q., Xu X., Yang Y., Zhen Y., Zhang Y., Zhou H., Fang H. (2022). Forythoside B ameliorates diabetic cognitive dysfunction by inhibiting hippocampal neuroinflammation in ovariectomized mice. Front. Aging Neurosciences.

[53] Kingsberg S.A., Larkin L.C., Liu J.H. (2020). Clinical effects of early or surgical menopause. OBGYN.

[54] Zhou Y., Xu B., Yu H., Zhao W., Song X., Liu Y., Wang K., Peacher N., Zhao X., Zhang H.T. (2021). Biochanin a attenuates ovariectomy-induced cognition deficit via antioxidant effects in female rats. Front. Pharmacol..

[55] W. R. (1997). Markesbery Oxidative stress hypothesis in Alzheimer's disease. Free Radic. Biol. Med..

[56] Van der Goes A., Brouwer J., Hoekstra K., Roos D., Van den Berg T.K., Dijkstra C.D. (1998). Reactive oxygen species are required for the phagocytosis of myelin by macrophages. J. Neuroimmunol..

[57] Jiang F., Zhang Y., Dusting G.J. (2011). NADPH oxidase-mediated redox signaling: roles in cellular stress response, stress tolerance, and tissue repair. Pharmacol. Rev..

[58] Coso S., Harrison I., Harrison C.B., Vinh A., Sobey C.G., Drummond G.R., Williams E.D., Selemidis S. (2012). NADPH oxidases as regulators of tumor angiogenesis: current and emerging concepts. Antioxid. Redox Signal..

[59] Zhu W., Oteiza P.I. (2023). NADPH oxidase 1: a target in the capacity of dimeric ECG and EGCG procyanidins to inhibit colorectal cancer cell invasion Redox. Biol..

[60] Welles E.K., Fridovich I. (1975). Superoxide, hydrogen peroxide, and singlet oxygen in lipid peroxidation by a xanthine oxidase system. JBC.

[61] Yousefian M., Shakour N., Hosseinzadeh H., Hayes A.W., Hadizadeh F., Karimi G. (2019). The natural phenolic compounds as modulators of NADPH oxidases in hypertension. Phytomedicine.

[62] Monteleone P., Mascagni G., Giannini A., Genazzani A.R., Simoncini T. (2018). Symptoms of menopause - global prevalence, physiology and implications. Nat. Rev. Endocrinol..

[63] Nelson B.S., Black K.L., Daniel J.M. (2016). Circulating estradiol regulates brain-derived estradiol via actions at GnRH receptors to impact memory in ovariectomized rats. eNeuro.

[64] Rauca C., Zerbe R., Jantze H., Krug M. (2000). The importance of free hydroxyl radicals to hypoxia preconditioning. Brain Res..

[65] Møller P., Loft S., Lundby C., Olsen N.V. (2001). Acute hypoxia and hypoxic exercise induce DNA strand breaks and oxidative DNA damage in humans. Faseb. J..

[66] An-Gaëlle G., Zgavc T., Kooijman R., Hachimi-Idrissi S., Sarre S., Michotte Y. (2010). The dual role of the neuroinflammatory response after ischemic stroke: modulatory effects of hypothermia. J. Neuroinflammation.

[67] Hiba H., Lutfi, G M.F., Sharara Saeed G.M.A.M. (2015). Oxidative/nitrosative stress in rats subjected to focal cerebral ischemia/reperfusion. Int. J. Health Sci..

[68] Juan F., Cueto-Escobedo J., Puga-Olguín A., Rivadeneyra-Domínguez E., Bernal-Morales B., Herrera-Huerta E.V., Santos-Torres A. (2017). The phytoestrogen genistein produces similar effects as 17β-estradiol on anxiety-like behavior in rats at 12 weeks after ovariectomy. BioMed Res. Int..

[69] Dowlati Y., Herrmann N., Swardfager W., Liu H., Sham L., Reim E.K., Lanctôt K.L. (2010). A meta-analysis of cytokines in major depression. Biol. Psychiatry..

[70] Wuwongse S., Chang R.C., Law A.C. (2010). The putative neurodegenerative links between depression and Alzheimer's disease. Prog. Neurobiol.

[71] Salim S., Chugh G., Asghar M. (2012). Inflammation in anxiety. Adv. Protein Chem. Struct. Biol..

[72] Gomez C.R., Plackett T.P., Kovacs E.J. (2007). Aging and estrogen: modulation of inflammatory responses after injury. Exp. Gerontol..

[73] Simi A., Lerouet D., Pinteaux E., Brough D. (2007). Mechanisms of regulation for interleukin-1 beta in neurodegenerative disease. Neuropharmacol.

[74] Lammers C.H., Schweitzer P., Facchinetti P., Arrang J.M., Madamba S.G., Siggins G.R., Piomelli D. (1996). Arachidonate 5-lipoxygenase and its activating protein: prominent hippocampal expression and role in somatostatin signaling. J. Neurochem..

[75] Chinnici C.M., Yao Y., Praticò D. (2007). The 5-lipoxygenase enzymatic pathway in the mouse brain: young versus old. Neurobiol. Aging.

[76] Czapski G.A., Czubowicz K., Strosznajder R.P. (2012). Evaulation of the antioxidative properties of lipoxygenase inhibitors. Pharmacol. Rep..

[77] Czubowicz K., Czapski G.A., Cieślik M., Strosznajder R.P. (2010). Lipoxygenase inhibitors protect brain cortex macromolecules against oxidation evoked by nitrosative stress. Folia Neuropathol..

[78] Kanaoka Y., Boyce J.A. (2014). Cysteinyl leukotrienes and their receptors: emerging concepts. Allergy Asthma Immunol. Res..

[79] Lüth H.J., Holzer M., Gärtner U. (2001). Expression of endothelial and inducible NOS-isoforms is increased in Alzheimer's disease, in APP23 transgenic mice and after experimental brain lesion in rat: evidence for an induction by amyloid pathology. Brain Res..

[80] Harooni H.E., Naghdi N., Sepehri H., Rohani A.H. (2009). The role of hippocampal nitric oxide (NO) on learning and immediate, shortand long-term memory retrieval in inhibitory avoidance task in male adult rats. Behav. Brain Res..

[81] Bandookwala M., Sengupta P. (2020). 3-Nitrotyrosine: a versatile oxidative stress biomarker for major neurodegenerative diseases. Int. J. Neurosci..

[82] Bourgognon J.M., Spiers J.G., W S. (2021). Robinson Inhibition of neuroinfammatory nitric oxide signaling suppresses glycation and prevents neuronal dysfunction in mouse prion disease. Proc. Natl. Acad. Sci. U.S.A..

[83] Kummer M.P., Hermes M., Delekarte A. (2011). Nitration of tyrosine 10 critically enhances amyloid b aggregation and plaque formation. Cell Press.

[84] Goh Y.X., Jalil J., Lam K.W., Husain K., Premakumar C.M. (2022). Genistein: a review on its anti-inflammatory properties. Front. Pharmacol..

[85] Liu H., Wu X., Luo J., Wang X., Guo H., Feng D., Zhao L., Bai H., Song M., Liu X., Guo W., Li X., Yue L., Wang B., Qu Y. (2019). Pterostilbene attenuates astrocytic inflammation and neuronal oxidative injury after ischemia-reperfusion by inhibiting NF-κB phosphorylation. Front. Immunol..

[86] Zohreh G., Basir Z., Jamshidian J., Delfi F. (2020). Modulation of behavioral responses and CA1 neuronal death by nitric oxide in the neonatal rat's hypoxia model. Brain Behav.

[87] Tominaga K., Yamauchi A., Shuto H., Niizeki M., Makino K., Oishi R., Kataoka Y. (2001). Ovariectomy aggravates convulsions and hippocampal gamma-aminobutyric acid inhibition induced by cyclosporin A in rats. Eur. J. Pharmacol..

[88] Zárate A., Fonseca E., Ochoa R., Basurto L., Hernández M. (2002). Low-dose conjugated equine estrogens elevate circulating neurotransmitters and improve the psychological well-being of menopausal women. Fertil. Steril..

[89] Rodríguez-Landa J.F., Puga-Olguín A., Germán-Ponciano L.J., García-Ríos R.-I., Soria-Fregozo C. (2015).

[90] Liu X., Song M., Chen X., Sun Y., Fan R., Wang L., Lin W., Hu Z., Zhao H. (2022). Activation of estrogen receptor β in the lateral habenula improves ovariectomy-induced anxiety-like behavior in rats. Front. Behav. Neurosci..

[91] Giannini A., Caretto M., Genazzani A.R., Simoncini T. (2021). Neuroendocrine changes during menopausal transition. Endocrines.

[92] Cynthia L.W.S., Smith A.W., Centeno M.L., Reddy A.P. (2011). Long-term ovariectomy decreases serotonin neuron number and gene expression in free ranging macaques. Neuroscience.

[93] Ganiyu O., Oladun F.L., Ademosun A.O., Ogunsuyi O.B. (2021). Anticholinesterase activity and antioxidant properties of *Heinsia crinita* and *Pterocarpus soyauxii* in *Drosophila melanogaster* model. J-AIM.

[94] Haam J., Yakel J.L. (2017). Cholinergic modulation of the hippocampal region and memory function. J. Neurochem..

[95] Santos-Galduróz Galduróz R.F., Facco J.C.F., Hachul H., Tufik S. (2010). Effects of isoflavone on the learning and memory of women in menopause: a double-blind placebo-controlled study. Braz. J. Med. Biol. Res..

